# Protease-activated CendR peptides targeting tenascin-C: mitigating off-target tissue accumulation

**DOI:** 10.1007/s13346-024-01670-2

**Published:** 2024-07-16

**Authors:** Allan Tobi, Maarja Haugas, Kristina Rabi, Jhalak Sethi, Kristina Põšnograjeva, Päärn Paiste, Toomas Jagomäe, Karlis Pleiko, Prakash Lingasamy, Tambet Teesalu

**Affiliations:** 1 Laboratory of Precision and Nanomedicine, Institute of Biomedicine and Translational Medicine, University of Tartu, Ravila 14B, 50411 Tartu, Estonia; 2 Department of Geology, Institute of Ecology and Earth Sciences, University of Tartu, Ravila 14A, 50411 Tartu, Estonia; 3 Laboratory Animal Centre, Institute of Biomedicine and Translational Medicine, University of Tartu, Ravila 14B, 50411 Tartu, Estonia; 4 Competence Centre on Health Technologies, Teaduspargi 13, 50411 Tartu, Estonia; 5 Materials Research Laboratory, University of California, Santa Barbara, CA 93106 USA

**Keywords:** Tumor-homing peptides, Phage display, Neuropilin-1, Tenascin-C, Silver nanoparticles, Glioblastoma

## Abstract

To achieve precision and selectivity, anticancer compounds and nanoparticles (NPs) can be targeted with affinity ligands that engage with malignancy-associated molecules in the blood vessels. While tumor-penetrating C-end Rule (CendR) peptides hold promise for precision tumor delivery, C-terminally exposed CendR peptides can accumulate undesirably in non-malignant tissues expressing neuropilin-1 (NRP-1), such as the lungs. One example of such promiscuous peptides is PL3 (sequence: AGRGRLVR), a peptide that engages with NRP-1 through its C-terminal CendR element, RLVR.

Here, we report the development of PL3 derivatives that bind to NRP-1 only after proteolytic processing by urokinase-type plasminogen activator (uPA), while maintaining binding to the other receptor of the peptide, the C-domain of tenascin-C (TNC-C). Through a rational design approach and screening of a uPA-treated peptide-phage library (PL3 peptide followed by four random amino acids) on the recombinant NRP-1, derivatives of the PL3 peptide capable of binding to NRP-1 only post-uPA processing were successfully identified. In vitro cleavage, binding, and internalization assays, along with in vivo biodistribution studies in orthotopic glioblastoma-bearing mice, confirmed the efficacy of two novel peptides, PL3uCendR (AGRGRLVR↓SAGGSVA) and SKLG (AGRGRLVR↓SKLG), which exhibit uPA-dependent binding to NRP-1, reducing off-target binding to healthy NRP-1-expressing tissues. Our study not only unveils novel uPA-dependent TNC-C targeting CendR peptides but also introduces a broader paradigm and establishes a technology for screening proteolytically activated tumor-penetrating peptides.

**Graphical Abstract:**

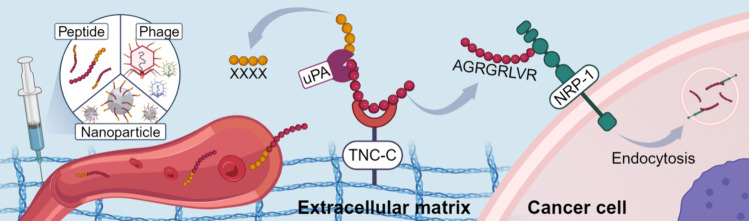

**Supplementary Information:**

The online version contains supplementary material available at 10.1007/s13346-024-01670-2.

## Introduction

Most anticancer drugs lack selectivity and have dose-limiting side effects [1]. Drug delivery is particularly challenging for hard-to-reach tumors such as pancreatic cancer and glioblastoma (GBM), the most prevalent and deadliest malignant primary brain tumor in adults [2, 3]. Challenges in tumor delivery have spurred the search for tumor-specific targets to enable targeted drug delivery [4]. Systemically accessible molecular heterogeneity in tumors includes angiogenesis or malignancy-associated changes on the surface of cells or in the composition of the extracellular matrix (ECM) [5, 6]. Affinity ligands can be used to target disease-associated molecular signatures in tumors to concentrate drugs and enhance therapeutic efficacy while reducing damage to healthy tissues [7, 8].

Tumor-homing peptides are used for targeted delivery of different classes of payloads, in particular nanomaterials, where even low-affinity ligands can dramatically enhance target-specific avidity through multivalent interactions [9]. Homing peptides are typically not species-specific as they engage functionally important binding pockets on target molecules, and these sites are conserved across species [10–12]. The potential of targeting peptides is illustrated by the translational efforts on the iRGD tumor-penetrating peptide (TPP) undergoing clinical development under the brand name LSTA1 for potentiating the delivery of anticancer therapeutics to pancreatic cancer and several other solid tumors [13].

TPPs are defined by the presence of a consensus R/KXXR/K motif able to bind to neuropilin-1 (NRP-1), a pleiotropic cell-surface receptor expressed in numerous normal organs and overexpressed in most solid tumors, including GBM [10, 14, 15]. Peptide interactions with NRP-1 activate a pathway for enhanced extravascular transport of conjugated and co-administered drugs [16, 17]. A defining characteristic of tumor-penetrating peptides and many of the natural ligands of NRP-1 (e.g., VEGFA165, Semaphorin-3) is that their specific interaction with NRP-1 is conditional and dependent on the C-terminal exposure of the R/KXXR/K motif, leading to its designation as the C-end Rule (CendR) motif [18–20]. This specificity arises from the peptide's mode of interaction with the receptor: CendR peptides utilize their C-terminal arginine residue to engage with the interloop cleft formed by the L3 loop (also known as the “C-wall”) and the L5 loop within the b1 domain of NRP-1 [21]. In the case of iRGD [22], LinTT1 [23] and iNGR [24] peptides, the CendR motif is cryptic and remains inactive until proteolytic cleavage in the tumor environment, whereas some other TPPs such as tLyP1 [25, 26] and PL3 [27] display C-terminal CendR motifs that do not require proteolytic activation. A drawback of the latter systems is the risk of off-target binding due to the expression of NRP-1 in healthy organs, particularly the lungs [28], potentially leading to adverse effects and compromised efficacy.

One strategy to mitigate off-target accumulation of TPPs with C-terminal CendR motifs involves concealing the CendR sequence with a tumor-specific protease cleavable motif, thereby generating cryptic CendR peptides [29]. Among the > 100 proteases associated with various aspects of cancer development and progression, the serine protease urokinase-type plasminogen activator (uPA) with its functionally related molecules, including receptors and inhibitors, represents one of the most well-established cases [30]. uPA cleaves the ubiquitous zymogen plasminogen at the amino acid bond Arg (561)–Val (562), resulting in the generation of the broad-spectrum serine protease plasmin [31]. Upregulated expression of uPA and its high-affinity receptor (uPAR) is associated with tumor progression, invasion, and metastasis; correlations discovered between uPA/uPAR expression and poor patient prognosis suggest that uPA levels could be used as a diagnostic or prognostic indicator [32–34]. Accordingly, the uPA/uPAR system has also been of interest as a potential drug target and activator of antitumor prodrugs [35]. To show the viability of the rational design approach for creating proteolytically activated CendR peptides for tumor targeting, we have previously engineered a uCendR peptide incorporating an optimal uPA cleavage site within the context of the CendR motif [36]. These proof-of-concept studies demonstrated that the uCendR peptide was effectively activated and exhibited robust in vivo tumor homing and penetration capabilities.

PL3 (sequence: AGRGRLVR) is a dual binding tumor-homing peptide [27]. Its primary target is a large modular extracellular matrix (ECM) glycoprotein tenascin-C (TNC) associated with fetal development and malignancy, and it also engages NRP-1 via its C-terminal CendR motif RLVR [27]. Tumor ECM is an aberrant meshwork of polymeric proteins and accessory molecules that forms the non-cellular component of solid tumors [37]. Tenascin-C (TNC) and especially its isoform targeted by PL3 peptide, the C-domain of TNC (TNC-C), shows low expression in non-malignant tissues and a robust overexpression in cancerous tissues, including GBM [38–40]. Compared to receptors expressed on the surface of malignant cells, TNC-C is primarily deposited by genetically stable stromal cells and may thus provide a more stable and high-capacity target for targeting ligands [40]. However, the presence of the active C-terminal CendR motif in PL3 may result in promiscuous accumulation in non-malignant tissues as observed for other non-cryptic CendR peptides [25, 26].

Here, we report the development of PL3 derivatives that contain cryptic CendR motifs which can be activated by exposure to uPA. In the quest for peptide discovery, we used both rational design and screening of combinatorial peptide libraries displayed on the T7 phage. Newly identified peptides showed uPA-dependent binding to NRP-1 and reduced off-target binding to healthy NRP-1-expressing pulmonary tissue. This study led to the discovery of novel uPA-dependent CendR peptides and introduces a broader paradigm for screening proteolytically activated tumor-penetrating peptides.

## Results and discussion

### Identification of uPA-cleavable derivatives of the PL3 peptide

Our demonstration of proteolytic activation of the CendR motif embedded within uPA recognition and cleavage sequences suggested the feasibility of proteolytically activated screening using constrained peptide libraries containing cryptic CendR motifs [36]. Such innovative approaches may be used for empirical identification of CendR peptides activated by various tumor-associated proteases, including those with poorly characterized substrate specificity.

**Fig. 1:**
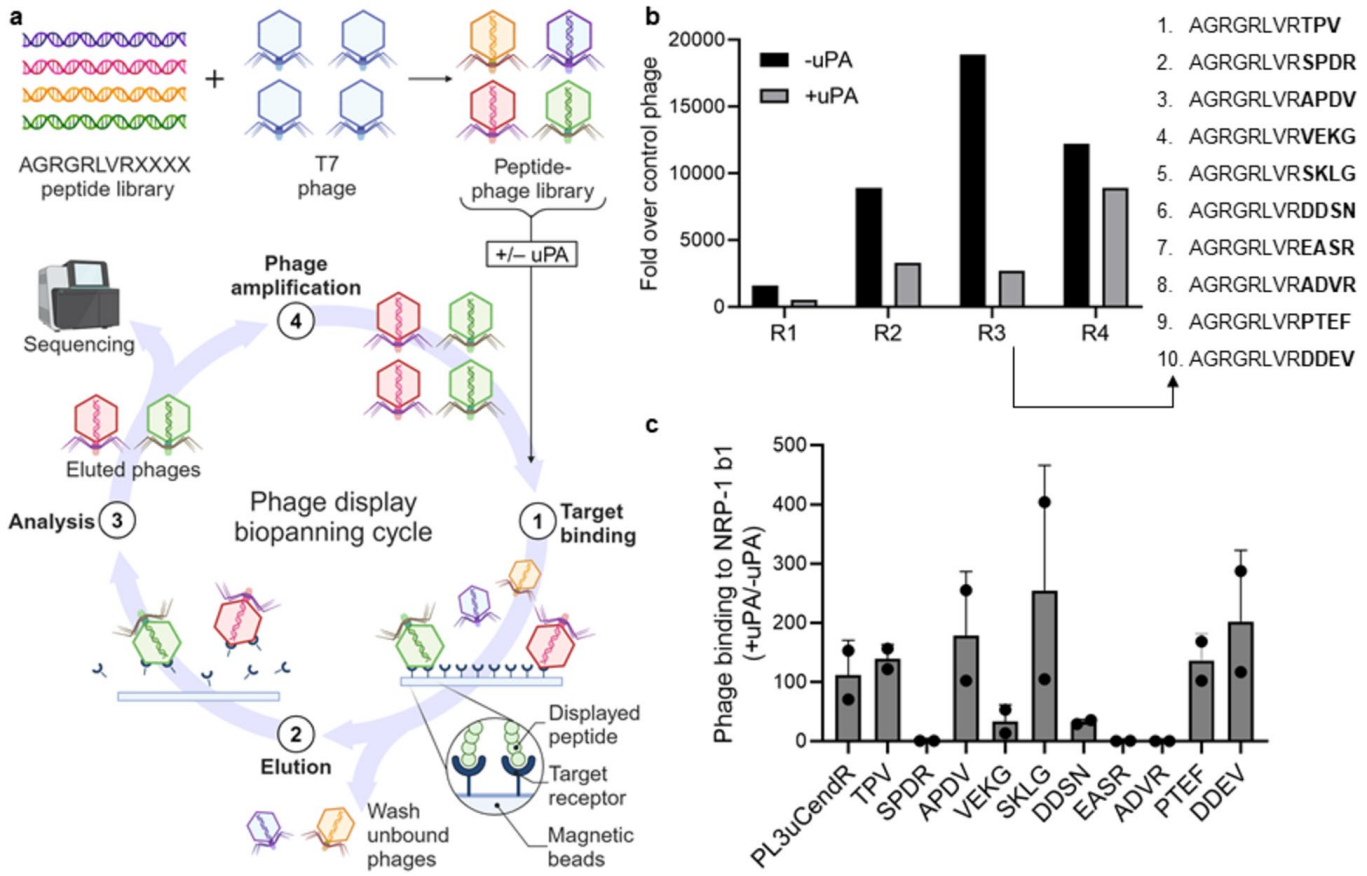
Discovery of uPA-dependent NRP-1 b1-binding PL3-derivative peptides. (**a**) Schematic of peptide-phage library design and biopanning screen. (**b**) Library enrichment through biopanning rounds 1–4 (R1–4). Ni–NTA magnetic agarose beads were coated with recombinant histidine-tagged b1 domain of NRP-1, incubated with phage pools with or without uPA pretreatment, washed, eluted, titered, and sequenced with HTS. Results are displayed as fold over control G7 peptide-phage (*n* = 1). Peptide sequences of top 10 phage candidates from round 3 based on + /– uPA enrichment ratios are listed. (**c**) Conditional NRP-1 b1 domain binding of individual phage candidates. Experiment was conducted as in (**b**), except individual phages were used and no HTS was performed. Names on the x-axis refer to the C-terminal part of the homing peptide on phage with reference to (**b**); PL3uCendR = AGRGRLVRSAGGSVA. Results are displayed as a ratio of + /– uPA normalized to control insertless phage. Error bars show standard deviation (SD), scatter symbols individual measurements (*n* = 2)

To validate the potential of such a screening approach in a proof-of-concept study, we conducted peptide-phage biopanning screens specifically targeting PL3-derived peptides sensitive to uPA. We constructed a peptide-phage library in which the PL3 sequence was C-terminally followed by four random amino acids (configuration: AGRGRLVRXXXX, where X is a random amino acid). For biopanning (Fig. 1a), the library was divided into two parallel pools. One pool was treated with uPA, followed by incubation with magnetic beads coated with the recombinant NRP-1 b1 domain to capture phages displaying conditionally active CendR peptides. The other pool was left non-treated to account for CendR peptides binding to the NRP-1 b1 domain regardless of uPA activation. The evolution of the peptide landscape throughout the screening was monitored by titering the bound phage and analyzing the peptide-encoding region of the phage genome by high throughput sequencing (HTS). HTS-based assessment of the representation of peptides in the third round of selection (Table S1) led to 10 candidate peptides with the highest relative enrichment in the uPA-treated pool (Fig. 1b), which were selected for individual testing. It should be noted that the phage pools without uPA pretreatment showed higher overall counts of bound phage, and a rise of overall peptide counts was seen in the uPA pretreated fraction of round 4 (Fig. 1b). The latter can be explained by the overrepresentation of R-ending sequences that bind NRP-1 regardless of uPA cleavage due to selective pressure, which prompted us to select round 3 for further analysis. Additionally, we designed a cryptic PL3 peptide that integrates a previously published uPA cleavage site compatible with CendR activation [36] (referred to as "PL3uCendR": AGRGRLVR↓SAGGSVA, where ↓ denotes the cleavage site).

Subsequently, the candidate peptide-phages were studied individually to determine the uPA-dependence of phage binding to recombinant NRP-1 b1 domain. After incubation with uPA, the phage displaying SKLG peptide exhibited a 255-fold mean increase in NRP-1 binding, while the PL3uCendR phage showed a 122-fold mean increase, ranking sixth among the tested peptide-phages (Fig. 1c). As anticipated, candidate peptides SPDR, EASR and ADVR, featuring C-terminal arginine residues, showed the highest NRP-1 binding in the absence of uPA treatment and the least enhancement following uPA treatment. Consequently, these peptides were excluded from further studies. Phages with SKLG and PL3uCendR peptides demonstrated retention of binding to TNC-C at levels comparable to the parent PL3 peptide (Fig. S1).

**Fig. 2:**
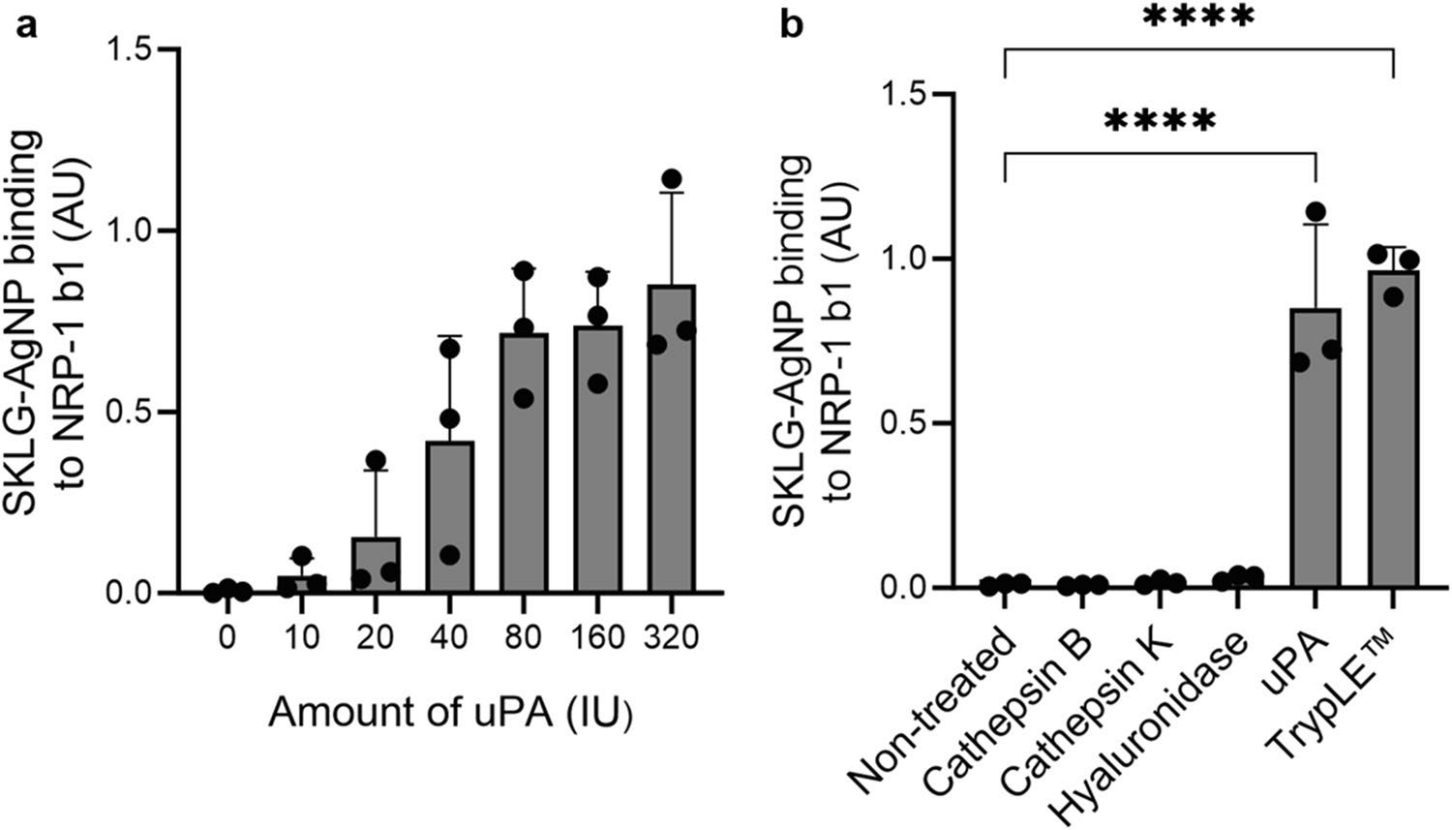
uPA treatment enhances binding of SKLG-AgNPs to NRP-1 b1 domain. (**a**) Dose-dependent cleavage of the SKLG peptide. Ni–NTA magnetic beads were coated with recombinant histidine-tagged b1 domain of NRP-1, incubated with SKLG-AgNPs, washed, and eluted. AgNP absorbance of bound SKLG-AgNPs was measured at 415 nm. AU = absorbance units; IU = international unit for enzymes; error bars show standard deviation (SD), scatter symbols individual measurements (*n* = 3). (**b**) uPA specifically cleaves the SKLG peptide at target site to induce NRP-1 b1 binding along with TrypLE™. Experiment was conducted as in (a). AU = absorbance units; IU = international unit for enzymes; error bars show standard deviation (SD), scatter symbols individual measurements (*n* = 3); **** = *p*-value < 0.0001, one-way ANOVA with Dunnett post-hoc

We next studied whether uPA-actuated increase in NRP-1 binding can be also observed for silver nanoparticles (AgNPs) functionalized with synthetic SKLG peptides. uPA pretreatment of AgNPs coated with the SKLG peptide (SKLG-AgNPs) activated the engagement of the nanoparticles with the recombinant b1 domain of NRP-1 in a uPA concentration-dependent manner (Fig. 2a). To study the specificity of the enzymatically actuated NRP-1 binding, the SKLG-AgNPs were pre-incubated with cathepsins B and K (cysteine proteinases), hyaluronidase (endoglycosidase), and a trypsin-like enzyme reagent TrypLE™ (serine proteinase), besides uPA. Cathepsins B and K as well as hyaluronidase failed to increase NRP-1 binding of the SKLG-AgNPs, whereas treatment with uPA and TrypLE™ resulted in robustly induced NRP-1 binding (Fig. 2b). Trypsin (and trypsin-like TrypLE™) is a broad-spectrum proteinase with cleavage specificity at the C-terminal side of basic amino acid residues, arginine and lysine [41]. Trypsin processing has been used to expose cryptic CendR elements in the past [22], and thus enhancement of binding of SKLG-AgNPs to NRP-1 was expected.

These studies demonstrated the feasibility of agnostic screening for proteolytically activated CendR peptides using cell-free selections on recombinant NRP-1 b1 domain and identified SKLG as a lead candidate peptide for uPA-mediated activation.

### Cellular uptake of nanoparticles equipped with PL3-derived cryptic CendR peptides is modulated by the expression of receptors and uPA cleavage

The specificity profile of each tumor-penetrating peptide is defined by an interplay of its primary recruitment receptor (e.g., in the case of iRGD the alpha v integrins [22], and in the case of LinTT1 cell surface receptor p32 [23]) and proteolysis-dependent engagement of the CendR element of a TPP with NRP-1.

To study the effect of cellular expression of peptide receptors on the binding of the PL3-derived cryptic CendR peptides, we performed a series of in vitro cell binding and internalization experiments on cells of known receptor expression status: U87-MG glioblastoma cells (TNC-C^+^, NRP-1^+^), PPC1 prostate cancer cells (TNC-C^–^, NRP-1^+^) and M21 melanoma cells (TNC-C^–^, NRP-1^–^) [27].

PL3, PL3uCendR and SKLG peptide-functionalized fluorescently CF555-labeled AgNPs were incubated with cultured cells and used in confocal microscopy-based experiments. The microscopy images of cellular binding studies reveal both the bound and internalized fraction of AgNPs, although herein referred to simply as “binding of AgNPs” for brevity. To differentiate only the fraction of internalized AgNPs, AgNP-treated live cells with an intact cell membrane were exposed to a biocompatible etching solution that dissolves extracellular AgNPs leaving intracellular nanoparticles intact [42]. Additionally, the nanoparticles were optionally subjected to uPA pretreatment to activate the cryptic CendR peptides.

**Fig. 3:**
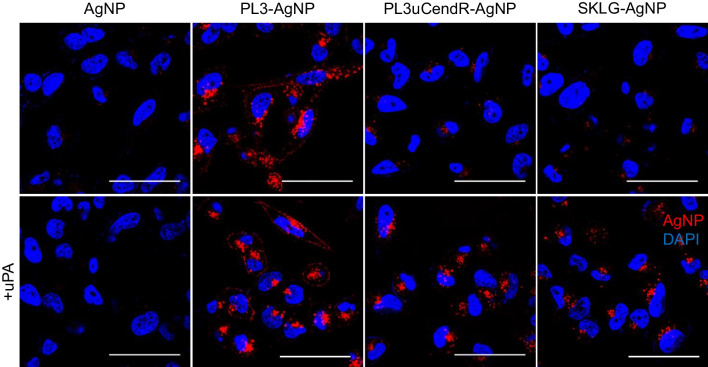
Internalization of CendR peptide-AgNPs in NRP-1-positive PPC1 cells. Prostate carcinoma (PPC1) cells were grown as a 2D culture, incubated with CF555-labeled AgNPs (red) optionally pretreated with uPA, washed, etched with a membrane-impermeable AgNP dissolving solution, fixed with –20 °C MeOH, counterstained with DAPI (blue; nuclei), and imaged. Representative images are shown (*n* = 3). Scale bar: 100 µm

We observed that whereas the PPC1 cells showed robust binding (Fig. S2) and internalization (Fig. 3) of parent PL3 peptide-functionalized AgNPs, the binding of PL3uCendR- and SKLG-AgNPs was dependent on uPA treatment, and no uptake was evident in the case of control AgNPs without peptide. In U87-MG cells, PL3-, PL3uCendR- and SKLG-AgNPs showed similar binding and internalization, whereas control AgNPs remained negative (Fig. S3). AgNPs decorated with PL3uCendR and SKLG peptides were retained by the U87-MG cells regardless of uPA treatment. We hypothesize that this may be due to turnover of TNC-C in these cells and processing by endogenous uPA, which was shown to be expressed in all tested cell lines (Fig. S4). Control M21 cells, negative for both TNC-C and NRP-1, did not bind nor internalize any of the tested AgNPs (Fig. S5).

These results show that cellular engagement of cryptic PL3 peptide-equipped AgNPs can be modulated by proteolytic switching via uPA. In NRP-1-high PPC1 cells, AgNPs functionalized with cryptic PL3 variants PL3uCendR and SKLG bound to and internalized in strictly uPA-dependent manner. In contrast, in TNC-C-expressing U87-MG cells, promiscuous binding was observed for both nanoparticles coated with parent PL3 peptide as well as its cryptic uPA-sensitive versions.

### SKLG- and PL3uCendR-phages show improved tissue distribution in GBM mice

**Fig. 4:**
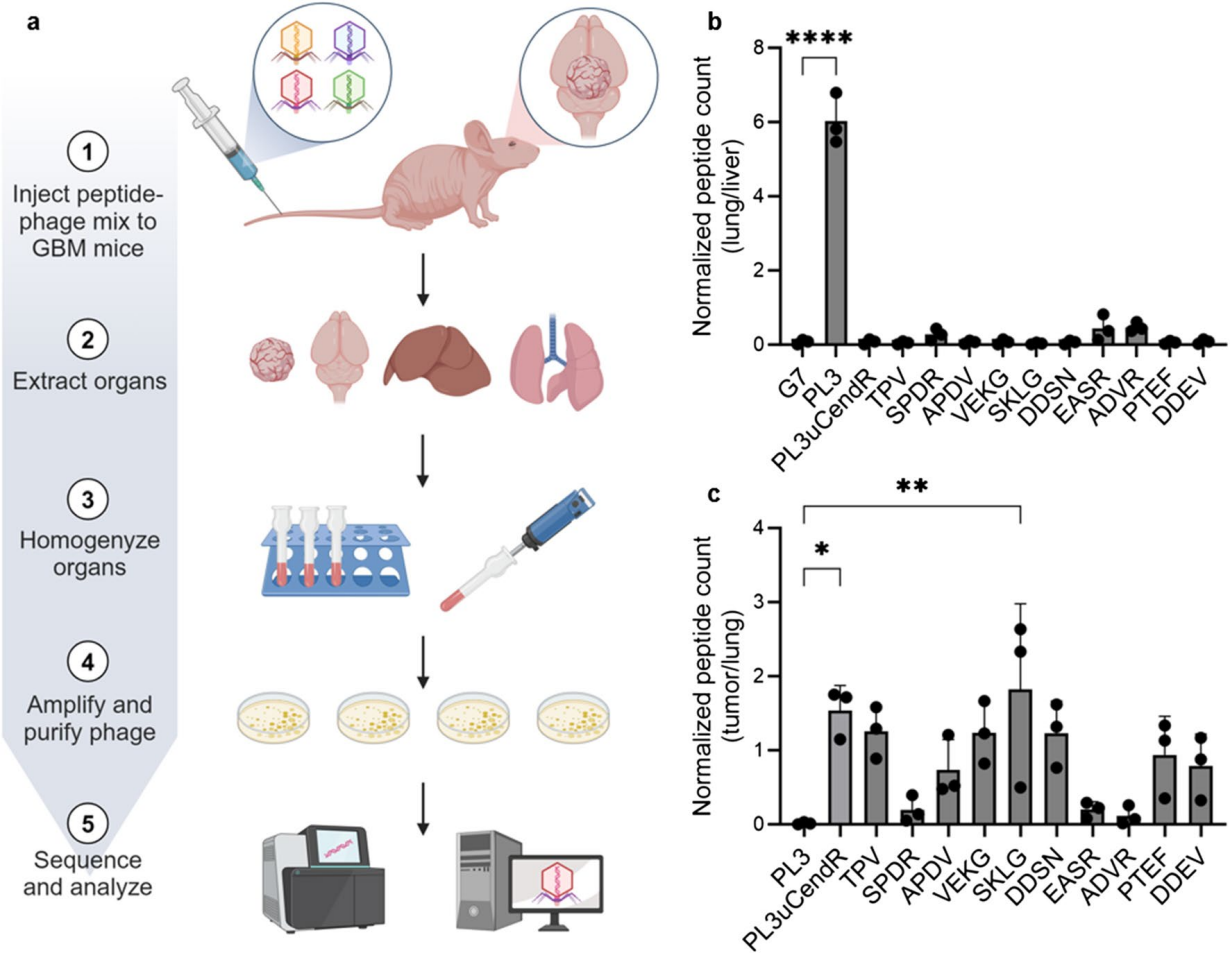
Auditioning candidate peptides by in vivo peptide-phage playoff. (**a**) Phages expressing peptide of interest or control peptide were mixed in an equimolar ratio and injected i.v. into WT-GBM-bearing female nude mice. After 30 min, mice were anesthetized and perfused, organs were harvested, homogenized, and tissue lysates were amplified, purified and sequenced with HTS. Results are shown as (**b**) peptide count in lung normalized to liver and (**c**) peptide count in WT-GBM tumor as a ratio of tumor over lung normalized to liver and control G7 phage. Error bars show standard deviation (SD), scatter symbols individual measurements (*n* = 3); * = *p*-value < 0.05, ** = *p*-value < 0.01, **** = *p*-value < 0.0001, one-way ANOVA with Dunnett post-hoc

Cell-free studies and cellular binding experiments described in preceding sections underscore the success in developing cryptic variants of the PL3 peptide capable of binding to NRP-1 following proteolytic processing by uPA. Given the low expression of uPA in normal adults, confined primarily to sites of physiological and pathological tissue remodeling [33], these peptides are not anticipated to exhibit promiscuous accumulation observed in the pulmonary and cardiac vascular beds, as seen with PL3 and other peptides bearing C-terminally exposed CendR motif, like RPARPAR [20, 36]. Nonetheless, systemic administration exposes nanoparticles and peptides to a milieu of factors absent in controlled cell-free/in vitro conditions—namely, plasma and tissue proteins, including a plethora of proteases and their regulatory molecules, alongside various other potentially interacting macromolecules. As these factors may affect the in vivo performance and specificity of the candidate peptides, we studied the biodistribution of the full panel of candidate peptides using in vivo peptide-phage playoff (Fig. 4a), a technique that allows comparative parallel evaluation of multiple candidate peptides in the same animal. An equimolar mixture of phages displaying 10 candidate peptides from the biopanning screen, PL3uCendR, PL3 or G7 negative control peptide was intravenously (i.v.) injected into athymic nude mice bearing orthotopically implanted SV40 large T-antigen and H-ras transformed mouse astrocytoma cell line (WT-GBM) xenografts expressing high levels of VEGF [43]. After 30 min of circulation the mice were perfused and retained phages in target and control tissues (tumor, brain, lungs, liver) were amplified and quantified with HTS.

In the lungs, PL3-phages were ~ 39 fold overrepresented compared to the cryptic candidate peptides (Fig. 4b). Phages displaying candidate peptides 2, 7 and 8 also showed slightly higher pulmonary accumulation, which can be attributed to the presence of C-terminal arginine in the sequence of these peptides. This observation aligns with prior research indicating that whereas the presence of a C-terminal R/KXXR/K is required for optimal NRP-1 binding activity, even a single arginine residue can confer binding [20, 44]. In brain tissue containing malignant lesions, the candidates exhibited modest accumulation (Fig. S6), possibly attributable to the highly angiogenic nature of WT-GBM tumors. These tumors are characterized by leaky blood vessels [45], which may lead to elevated baseline accumulation within the tumor.

When expressing the in vivo playoff data as a ratio of tumor over lung, SKLG and PL3uCendR were confirmed as the leading peptides with *p*-values of 0.0048 and 0.0274, respectively (Fig. 4c).

### Synthetic SKLG and PL3uCendR peptides show decreased pulmonary uptake in GBM mice while accumulating in the tumor

The previous section showed that the novel peptides decrease lung accumulation when displayed on the surface of biological nanoparticles, bacteriophages. We next evaluated the in vivo performance of the synthetic peptides (as monomeric peptides and conjugated to nanoparticles) in GBM mice.

We first stratified a panel of orthotopic glioblastoma models (WT-GBM, NCH421k, U87-MG, and VEGF-KO) for the expression of TNC-C, NRP-1 and uPA using immunostaining and confocal imaging (Fig. S7 and S8). Whereas clear upregulation (in comparison to normal brain tissue) of the immunoreactivities of TNC-C, NRP-1 and uPA was observed in WT-GBM, NCH421k and U87-MG tumor lesions, VEGF-KO tumors (generated by implantation of the VEGF-negative derivative subline of WT-GBM that gives rise to infiltrative nonangiogenic GBM [46]) expressed very low levels of uPA and were excluded from subsequent testing. In line with previous studies [14], some NRP-1 signal was also present in the tumor-free part of the brain. To study NRP-1 expression in the brain relative to the blood vessels, a colocalization study with anti-NRP-1 and anti-CD31 (blood vessel marker) antibodies was performed (Fig. S8). The blood vessels in the tumor-free part of the brain did not show any colocalization with NRP-1, indicating that the NRP-1 in the tumor-free brain regions is inaccessible upon i.v. administration, whereas colocalization was evident across the WT-GBM tumor (Fig. S﻿8, arrowheads). Confocal microscopy images of the tumor-free regions of the brains of tumor-bearing mice were surveyed to further ensure that neither the targeted nor control AgNPs accumulate in the tumor-free regions of the brain (Fig. S9).

**Fig. 5:**
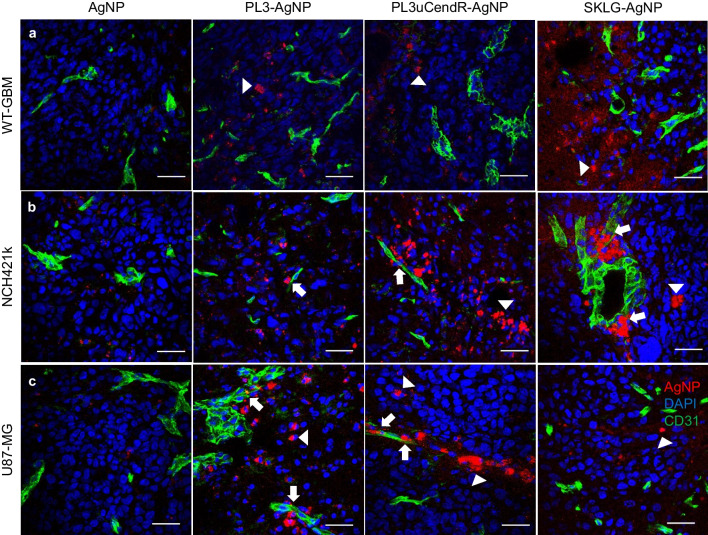
Biodistribution of peptide-AgNPs in (**a**) WT-GBM, (**b**) NCH421k and (**c**) U87-MG GBM models. Orthotopic GBM-bearing nude mice were i.v. injected with CF555-labeled AgNPs (red). After 3 h of circulation the mice were anesthetized and perfused. Organs were harvested, sectioned, immunostained with anti-CD31 antibody (green; blood vessels), counterstained with DAPI (blue; nuclei). Representative images are presented (*n* = 3). Arrows point to AgNPs in or near CD31-positive blood vessels, arrowheads to extravasated AgNPs. Scale bar: 100 µm

For biodistribution studies, GBM mice were dosed with AgNPs coated with either PL3, PL3uCendR or SKLG peptides. After circulation, the animals were perfused, and tissues (brain with tumor, lungs, liver) were collected, sectioned, and used for confocal microscopy imaging. Confocal microscopy imaging of CF555-labeled AgNPs decorated with targeting peptides showed that in all three tested tumor models the targeted AgNPs accumulated after 3 h of circulation near the tumor blood vessels and appeared to extravasate outside the CD31-positive vessels into the tumor parenchyma (Fig. 5). Although control AgNPs without targeting peptide seemed to accumulate less in the tumor tissue, areas with higher accumulation could still be seen, especially for the leaky WT-GBM and U87-MG models (Fig. S10).

In the pulmonary tissue, PL3-AgNPs showed higher accumulation than PL3uCendR-, SKLG- and control AgNPs (Fig. S11a). Hepatic tissue showed robust nonselective uptake of all types of AgNPs independent of the peptide functionalization (Fig. S11b).

Subsequently, we employed isotopically barcoded peptide-AgNPs and control AgNPs for internally-controlled laser ablation inductively coupled plasma mass spectrometry (LA-ICP-MS)-based quantitative mapping studies [11, 47, 48]. An equimolar mixture of ^109^AgNP-peptide and biotin-blocked control ^107^AgNPs was intravenously injected into orthotopic WT-GBM-bearing mice. Following tissue collection and sectioning, LA-ICP-MS was used to quantitatively map the 2D distribution of both types of NPs. In the lungs, PL3-^109^AgNPs exhibited approximately 2.5 times higher accumulation compared to co-injected control ^107^AgNPs. Conversely, for PL3uCendR- and SKLG-^109^AgNPs, the ^109^Ag/^107^Ag ratio in the lungs approached 1 (Fig. S12). These studies showed that the newly identified cryptic PL3 variants exhibit decreased off-target binding to healthy lungs.

The accumulation of PL3-, PL3uCendR- and SKLG-^109^AgNPs in tumors appeared similar to control ^107^AgNPs (Fig. S12). CendR peptides are known to trigger a bystander effect, activating bulk transport pathway to tumors, which could potentially limit the applicability of coadministration-based ratiometric biodistribution analyses [49]. Here, CendR peptide-^109^AgNPs may have triggered tumor transport of co-circulating control ^107^AgNPs. WT-GBM tumors are also highly angiogenic and contain dysfunctional and leaky neovessels [45], a factor that may have contributed to increased nonspecific retention of AgNPs in the tumor tissue.

Whereas polyvalent targeting systems, such as bacteriophages and AgNPs used in previous sections, exploit multivalent interactions to enhance specificity and binding efficiency, monovalent targeting systems rely on single ligand-receptor interactions for targeted drug delivery [50]. The choice between these two approaches depends on affinity of the targeting ligand and the pharmacokinetic properties of the therapeutic agent. We next studied in vivo biodistribution of monomeric FAM-labeled SKLG, PL3, and scrambled PL3 control (scrPL3) peptides in WT-GBM tumor-bearing mice. The mice were dosed with FAM-labeled peptides followed by perfusion, tissue collection, sectioning, and detection of the peptides using immunohistochemistry with anti-FAM antibodies.

**Fig. 6:**
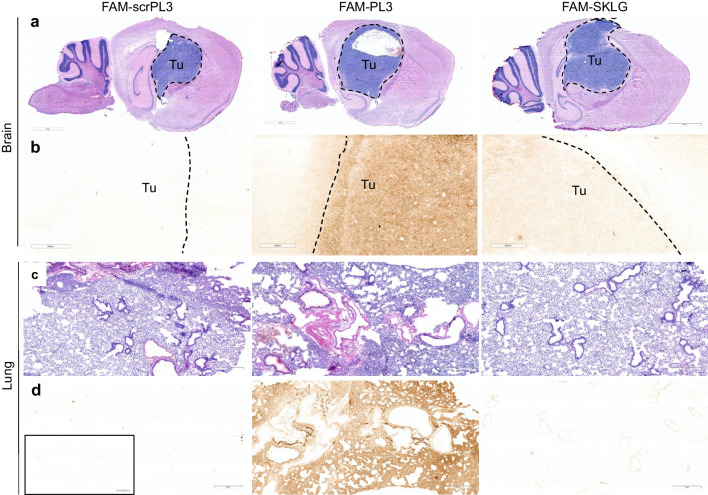
Biodistribution of monomeric FAM-labeled peptides in WT-GBM mice. Orthotopic GBM-bearing nude mice were i.v. injected with FAM-labeled scrambled PL3 control (scrPL3), PL3 or SKLG peptides. After 1 h of circulation the mice were anesthetized and perfused. Organs were harvested, sectioned, and parallel sections were immunostained with hematoxylin & eosin (H&E) staining or anti-FAM 3, 3'-diaminobenzidine (DAB) staining. (**a**) H&E of WT-GBM tumor-bearing brain tissue sections. (**b**) DAB staining of WT-GBM tumor region in the brain. (**c**) H&E staining of lung tissue sections. (**d**) DAB staining of lung tissue sections. Representative images are presented (*n* = 3). Dashed lines outline tumor; Tu = tumor. Insert in (**d**) shows secondary antibody control for DAB staining. Scale bars: 3 mm for (**a**), 500 µm for (**b**), 1 mm for (**c**, **d**)

In pulmonary tissue, a robust and consistent accumulation of the PL3 peptide was evident, whereas cryptic SKLG and scrambled PL3 control peptides exhibited only a background signal comparable to that of the secondary antibody alone (Fig. 6d insert). Tumor signal does show somewhat ambiguous preferential accumulation for PL3 and SKLG peptides compared to scrambled PL3 (scrPL3) control peptide (Fig. 6). Although the employed method of detection is qualitative rather than quantitative, it does seem that the SKLG peptide exhibits a lower signal compared to the PL3 peptide, which may suggest that the SKLG peptide is better suited for multivalent targeting approaches.

These findings align with the phage- and nanoparticle-based data previously presented, and underscore the importance of minimizing off-target binding of non-cryptic CendR-peptides, particularly in the lungs.

## Conclusion

We demonstrated that cell-free peptide-phage display can be used to identify novel proteolytically activated tumor-penetrating peptides. Two PL3 peptide derivatives, PL3uCendR (sequence: AGRGRLVRSAGGSVA) and SKLG (sequence: AGRGRLVRSKLG), that showed uPA-dependent activation of binding to cell-free and cell surface-expressed NRP-1 were identified in this study. In vivo, both monomeric and nanoparticle-tethered uPA-actuated PL3-derivative peptides showed glioblastoma homing and a robust reduction of pulmonary accumulation. The potential for targeting anticancer drugs with cryptic PL3 derivatives should be further investigated in future studies to determine if efficacious drug concentrations in the tumor can be achieved while avoiding side effects.

We envision the application of this system not only for modifying existing dual-binding homing peptides plagued by off-target accumulation but also discovering fully new target receptor binding protease-activated peptides where the exact cleavage site may be initially unknown. The overexpression of various proteases in glioblastomas or other types of solid tumors enables the application of such protease-sensitive peptide targeting moieties for targeted delivery of cargo molecules and nanosystems.

## Materials and methods

### Phage cloning

Nucleotide sequences encoding candidate peptides were cloned into T7 415-1b phage (Novagen, EMD Biosciences, USA) genomic DNA to be expressed on the phage surface as C-terminal fusions to capsid protein. The cloning was performed using complementary oligonucleotides (Integrated DNA Technologies, USA) as shown in the following Table 1. Complementary oligonucleotides were diluted in milli-Q (MQ) water at 100 nM, heated at 80 °C for 10 min, and slowly cooled to room temperature for annealing. The cloning was performed according to the manufacturer's protocol using the T7Select® 415–1 Cloning Kit (#70,015–3, Millipore) with 1 μL of annealed oligonucleotides.

**Table 1:** Complementary oligos for peptide-phage cloning

Peptide sequence	Sense oligomer	Antisense oligomer
AGRGRLVRTPV	AATTCTGCGGGCCGCGGCCGCCTGGTGCGCACCCCGGTGTA	AGCTTACACCGGGGTGCGCACCAGGCGGCCGCGGCCCGCAG
AGRGRLVRSPDR	AATTCTGCGGGCCGCGGCCGCCTGGTGCGCAGCCCGGATCGCTA	AGCTTAGCGATCCGGGCTGCGCACCAGGCGGCCGCGGCCCGCAG
AGRGRLVRAPDV	AATTCTGCGGGCCGCGGCCGCCTGGTGCGCGCGCCGGATGTGTA	AGCTTACACATCCGGCGCGCGCACCAGGCGGCCGCGGCCCGCAG
AGRGRLVRVEKG	AATTCTGCGGGCCGCGGCCGCCTGGTGCGCGTGGAAAAAGGCTA	AGCTTAGCCTTTTTCCACGCGCACCAGGCGGCCGCGGCCCGCAG
AGRGRLVRSKLG	AATTCTGCGGGCCGCGGCCGCCTGGTGCGCAGCAAACTGGGCTA	AGCTTAGCCCAGTTTGCTGCGCACCAGGCGGCCGCGGCCCGCAG
AGRGRLVRDDSN	AATTCTGCGGGCCGCGGCCGCCTGGTGCGCGATGATAGCAACTA	AGCTTAGTTGCTATCATCGCGCACCAGGCGGCCGCGGCCCGCAG
AGRGRLVREASR	AATTCTGCCGGCAGGGGCAGGCTGGTGAGGGAGGCCAGCAGGTA	AGCTTACCTGCTGGCCTCCCTCACCAGCCTGCCCCTGCCGGCAG
AGRGRLVRADVR	AATTCTGCGGGCCGCGGCCGCCTGGTGCGCGCGGATGTGCGCTA	AGCTTAGCGCACATCCGCGCGCACCAGGCGGCCGCGGCCCGCAG
AGRGRLVRPTEF	AATTCTGCGGGCCGCGGCCGCCTGGTGCGCCCGACCGAATTTTA	AGCTTAAAATTCGGTCGGGCGCACCAGGCGGCCGCGGCCCGCAG
AGRGRLVRDDEV	AATTCTGCGGGCCGCGGCCGCCTGGTGCGCGATGATGAAGTGTA	AGCTTACACTTCATCATCGCGCACCAGGCGGCCGCGGCCCGCAG
AGRGRLVR	AATTCTGCGGGCCGCGGACGTTGGTGCGCTA	AGCTTAGCGCACCAAACGTCCGCGGCCCGCAG
AGRGRLVRSAGGSVA	AATTCTGCTGGGCGCGGCCGTTTAGTGCGCTCAGCAGGGGGGTCTGTTGCTTA	AGCTTAAGCAACAGACCCCCCTGCTGAGCGCACTAAACGGCCGCGCCCAGCAG
AGRGRLVRXXXX	AATTCTGCGGGCCGCGGACGTTGGTGCGCNNKNNKNNKNNKTA	AGCTTAMNNMNNMNNMNNGCGCACCAAACGTCCGCGGCCCGCAG

X = random amino acid

### Phage amplification and purification

As described by Põšnograjeva et al. [51], 20 mL of LB/carbenicillin medium (Sigma-Aldrich, USA) was inoculated with a single colony of *E. coli* strain BLT5615 (Novagen, EMD Biosciences) from a freshly streaked LB/ampicillin (Sigma-Aldrich, USA) agar (Neogen, USA) plate (Nerbe Plus, Germany). The culture was incubated in a 100 mL Erlenmeyer flask (Simax, Czech Rebuplic) in a bacterial shaker (Innova 43, New Brunswick Scientific, USA) at 37 °C and 200 rpm overnight. Next morning, culture was diluted 1: 100 in pre-warmed LB/carbenicillin (Sigma-Aldrich, USA) and incubated in the shaker at 37 °C and 200 rpm until OD_600_ = 0.5.

For preamplification, 1 mL of BLT5403 (OD_600_ = 0.1) was inoculated with 10 μL of phage in Dulbecco’s Phosphate Buffered Saline (DPBS; PAN Biotech, Germany) in 50 mL Falcon tubes (Sigma-Aldrich, USA) in a shaker at 37 °C and 200 rpm for 1 h. Next, 25 mL of BLT5403 (OD_600_ = 0.5) was added.

For amplification, bacteria were inoculated with phage stock at a multiplicity of infection 0.001–0.01, and incubated in a shaker at 37 °C and 200 rpm for 1.5 h until solution became clear. The lysate was cooled on ice and divided into 50 mL Falcon tubes (Sigma-Aldrich, USA), 26 mL of lysate per tube. To each tube, 3 mL of 5 M NaCl (Sigma-Aldrich, USA) was added, and mixed by vortexing. Lysate was cleared of bacterial debris by centrifugation at 12,000 × *g* and 4 °C for 10 min. Supernatant was carefully transferred into a new 50 mL tube. Phage was precipitated with 8.4 mL of 50% PEG8000 (Sigma-Aldrich, USA) in Phosphate Buffered Saline (PBS; Sigma-Aldrich, USA). The mixture was vortexed and incubated on ice for at least 30 min. Precipitated phage was pelleted by centrifugation at 8,000 × *g* and 4 °C for 10 min. Supernatant was discarded and the tubes were air-dried upside-down on a paper towel for 15 min. Phage pellet was resuspended in 1.5 mL of PBS.

For density gradient purification, 62.5% CsCl (Sigma-Aldrich, USA) solution in PBS was used to prepare 2: 1, 1: 1, 1: 2 dilutions of CsCl : PBS, which were sequentially pipetted in the given order into a ultracentrifugation tube (0.25, 1.2, 1.2, 0.25 mL, respectively). On top of the CsCl gradient, 1.5 mL of PEG-purified phage was added. Tubes were centrifuged with a SW 60 Ti swing-out rotor (Beckman Coulter) at 40,000 rpm at 22 °C for 45 min. Phages form a light blue band, which was collected by puncturing the tube with a 21G needle and using a 1 mL syringe. Phage sample was dialyzed with a 3,500 MW cut-off Slide-A-Lyzer cassette (#66,330, Thermo Fisher, USA) against PBS at RT for 1 h and again 1 h after changing the buffer.

### Phage titering

As described by Põšnograjeva et al. [51], for titering T7 415-1b phage (Novagen, EMD Biosciences, USA), 20 mL of LB/carbenicillin medium (Sigma-Aldrich, USA) was inoculated with a single colony of *E. coli* strain BLT5615 (Novagen, EMD Biosciences, USA) from a freshly streaked LB/ampicillin (Sigma-Aldrich, USA) agar (Neogen, USA) plate (Nerbe Plus, Germany). The culture was incubated in a 100 mL Erlenmeyer flask (Simax, Czech Rebuplic) in a bacterial shaker (Innova 43, New Brunswick Scientific, USA) at 37 °C and 200 rpm overnight. Next morning, culture was diluted 1: 100 in pre-warmed LB/carbenicillin and incubated in the shaker at 37 °C and 200 rpm until OD_600_ = 0.5. Serial dilutions of phage in LB/carbenicillin were prepared and 100 μL of diluted phage in 15 mL Falcon tubes (Sigma-Aldrich, USA) was combined with 500 μL of BLT5615 culture. To each 15 mL tube, 5 mL of 50–60 °C top-agar containing 2 mM IPTG (Thermo Fisher, USA) was added. Tubes were vortexed and immediately plated on LB/ampicillin (Sigma-Aldrich, USA) agar (Neogen, USA) plate (Nerbe Plus, Germany). Plates were incubated overnight at RT. Formed phage plaques were visually counted, and phage concentration in the undiluted sample was calculated.

### Cell-free phage display

To screen for uPA-cleavable cryptic PL3 peptide variants, we constructed a constrained phage library (configuration: AGRGRLVRXXXX, where X is a random amino acid). The phage library was cloned as described above, using partially randomized oligos (Table 1). Generated peptide-phage library pool was divided into two groups: non-treated and uPA pretreated. See below for uPA treatment protocol.

For cell-free phage display, 2 mg/mL of hexahistidine-tagged recombinant b1 domain of NRP-1 (in-house) was coated onto Ni–NTA magnetic agarose beads (#36,113, Qiagen GmbH, Hilden, Germany) by incubating with end-over-end mixing at RT for 1–1.5 h. Tris buffer (50 mM, pH 7.0) containing 5 mM imidazole, 1 M NaCl and 0.05% Igepal CA-630 (all from Thermo Scientific Inc.) was used for the dilutions; the same buffer with 0.1% bovine serum albumin (BSA; GE Healthcare, Little Chalfont, UK) was used for washes after the protein coating step. Protein-coated magnetic beads were incubated with phage library pools (5 × 10^9^ pfu/mL) with end-over-end mixing for 1 h, followed by six washes with the washing buffer, and the release of protein–bound fraction with imidazole elution buffer (400 mM imidazole, 300 mM NaCl, 0.1% BSA and 0.05% Igepal CA-630 in PBS). Eluted phages were titered as described above.

### In vivo phage playoff

The HTS-based phage in vivo playoff method allows comparative internally controlled auditioning of tissue selectivity of systemic candidate peptide-phages. Phages expressing candidates 1–10, PL3uCendR (AGRGRLVRSAGGSVA), PL3 (AGRGRLVR) or G7 (CGGGGGGGC) peptides were amplified in *E. coli* strain BLT5403 (Novagen, EMD Biosciences), mixed in an equimolar ratio, purified by PEG8000 (Sigma-Aldrich, USA) precipitation, followed by CsCl gradient centrifugation, and dialyzed against PBS. 5 × 10^9^ pfu of phage mix was injected i.v. into orthotopic WT-GBM-bearing female nude mice (*n* = 3). After 30 min, the animals were anesthetized and perfused with PBS. The target and control organs (brain, tumor, lung, liver) were collected, the tissues were homogenized and phages in tissue lysates were amplified and purified by PEG8000 precipitation. The distribution of phages between target and control organs was determined by HTS as described below. Fold change of peptide count in an organ of interest was calculated according to the following general formula:

$$\text{Fold change}=\frac{{\text{pep}}_{\text{organ}}/{\text{pep}}_{\text{input}}}{\text{G}{7}_{\text{organ}}/\text{G}{7}_{\text{input}}}$$

where

pep_organ_ = % of reads coming from the peptide of interest in particular organ sequencing dataset;

pep_input_ = % of reads coming from the peptide of interest in input library sequencing dataset;

G7_organ_ = % of reads coming from control G7 peptide in particular organ sequencing dataset;

G7_input_ = % of reads coming from control G7 peptide in input library sequencing dataset.

## Sequencing

As previously described by Pleiko et al. [52], peptide-encoding region of bacteriophage genome was amplified by PCR using Phusion Green Hot Start II High-Fidelity DNA Polymerase (#F537L, Thermo Scientific) in 25 μL reaction volume. Cycling conditions: denaturation at 98 °C for 30 s, followed by 25 amplification cycles (10 s at 98 °C, 21 s at 72 °C), and final elongation (72 °C for 5 min). Polymerase chain reaction (PCR) products were purified using AMPure XP Bead Based Next-Generation Sequencing Cleanup system (Beckmann Coulter, A63881) using 1.8 μL of beads per 1 μL of PCR products. Purified PCR products were quantified using Agilent Bioanalyzer 2100 Instrument using the High sensitivity DNA Kit (#5067–4626, Agilent). Ion Torrent Emulsion PCR and enrichment steps were performed using Ion PGM HiQ View OT2 kit (#A29900, Life Technologies). High throughput sequencing (HTS) was performed using Ion Torrent™ Personal Genome Machine™ (ION-PGM) using Ion PGM HiQ View sequencing kit (#A30044, Life Technologies) and Ion 316v2 chips (#448,149, Life Technologies). The FASTQ sequence files were converted to text files and translated using in-house developed python scripts.

### Enzyme/uPA pretreatment

Per 50 µL of peptide-AgNPs (5.6 nM) or 50 µL of phage (1 × 10^9^ pfu/mL) 2–32 µL of uPA (10,000 U/mL; #672,112, Sigma-Aldrich) was added and incubated at 37 °C for 1 h.

In enzymatic cleavage specificity experiments, AgNPs were treated with 2 mg/mL of cathepsin B (#SRP0289, Sigma-Aldrich), 1.3 mg/mL cathepsin K (in-house), 1 mg/mL hyaluronidase (#H3884, Sigma-Aldrich) or 1X TrypLE™ Express (#12,604–019, Gibco) in addition to 32 µL of uPA (10,000 U/mL; #672,112, Sigma-Aldrich) in the same experimental conditions.

### Cell-free AgNP binding experiments

Cell-free binding studies were done as described in chapter 4.4 “Cell-free phage display”, except 2 mg/mL of hexahistidine-tagged recombinant b1 domain of NRP-1 (in-house) or C-domain of TNC (TNC-C; in-house) was used for coating. For NRP-1 b1, Tris buffer (50 mM, pH 7.0) containing 5 mM imidazole, 1 M NaCl and 0.05% Igepal CA-630 (all from Thermo Scientific Inc.), or for TNC-C and negative control NRP-1 b1 mutant (mut), PBS containing 0.05% Igepal CA-630 (Thermo Scientific Inc.), was used for the protein binding step. For incubations, 0.5 nM peptide-AgNPs were used, and eluted proteins with bound peptide-AgNPs were quantified by UV–vis spectrometry at 415 nm using a Nanodrop 2000c spectrophotometer (Thermo Scientific Inc.).

### Peptides

All synthetic peptides used herein were ordered as powder with a purity of > 95%, and reconstituted in PBS. Sequences of peptides are shown in Table 2 (all from TAG Copenhagen, Denmark).

**Table 2:** Synthetic peptides

Name	Sequence	MW (Da)
Biotin-PL3	Biotin-Ahx-AGRGRLVR	1223.49
Biotin-PL3uCendR	Biotin-Ahx-AGRGRLVRSAGGSVA	1753.04
Biotin-SKLG	Biotin-Ahx-AGRGRLVRSKLG	1608.95
FAM-PL3	5’-FAM-Ahx-AGRGRLVR	1458.64
FAM-scrPL3	5’-FAM-Ahx-RAGRGRLV	1355.50
FAM-SKLG	5’-FAM-Ahx-AGRGRLVRSKLG	1740.96

Ahx = aminohexanoic acid spacer

### Synthesis and functionalization of AgNPs

Silver nanoparticles were synthesized according to the Lee and Meisel citrate method [53]. Surface functionalization of AgNPs was done as previously published [42, 54]. Briefly, AgNO_3_ (360 mg; #209,139, Sigma-Aldrich Co., LLC, Darmstadt, Germany) or ^107^AgNO_3_ or ^109^AgNO_3_ (Isoflex USA, San Fransisco, CA, USA) was added to ultrapure Milli-Q (MQ) water (2 L; resistivity 18 MΩ cm^−1^) in a flask cleaned with a piranha solution (H_2_SO_4_/H_2_O_2_). Next, trisodium citrate hydrate (400 mg; #25,114, Sigma-Aldrich Co., LLC) was dissolved in MQ water (40 mL) and added to the vessel. The solution was boiled for 30 min in the dark. The resulting Ag-citrate was used directly in the next step.

Next, NeutrAvidin (NA; #31,055, Thermo Scientific Inc., Washington, USA) was modified with a OPSS-PEG(5 K)-SCM linker (OPSS; JenKem Technology USA Inc., Texas, USA) according to the procedure described by Braun et al. [42] Subsequently, NeutrAvidin-OPSS (3.9 mL, 2.9 mg/mL) was added to Ag-citrate (500 mL). After 2 min, 4-morpholineethanesulfonic acid hemisodium salt (5 mL, 0.5 M in MQ water; #M0164, Sigma-Aldrich Co., LLC) was added. The pH of the solution was adjusted to 6.0, and the solution was kept at 37 °C for 24 h. The solution was brought to room temperature (RT) and 10X phosphate buffered saline (50 mL; in-house) was added, followed by Tween® 20 (250 µL; #P9416, Sigma-Aldrich Co., LLC). The solution was centrifuged at 12,200 × g for 1 h at 4 °C, the supernatant was removed, and the particles were resuspended in PBST (0.005% Tween® 20 in PBS). Next, tris(2-carboxyethyl)phosphine hydrochloride solution (TCEP; #646,547, Sigma-Aldrich Co., LLC) was added to a final concentration of 1 mM, followed by a 30-min incubation at RT. Then, lipoic acid-PEG(1 k)-NH_2_ (#PG2-AMLA-1k, Nanocs Inc., New York, USA) was added to a final concentration of 5 µM, and the mixture was incubated at RT for 2.5 h. The solution was centrifuged at 17,200 × g for 20 min at 4 °C, the supernatant was removed, and the particles were resuspended in PBST to half of the initial volume. The AgNP solution was filtered through a 0.45 µm filter and stored at 4 °C.

NHS-functionalized CF555 dye (#92,130, Biotium Inc., California, USA) was coupled to the NH_2_ groups on the AgNPs. For this, NHS-CF555 (5 µL, 2 mM) in dimethyl sulfoxide (DMSO; Sigma-Aldrich Co., LLC) was added to AgNA (500 µL), followed by an overnight incubation at 4 °C. The particles were washed 3 times by centrifugation at 3,500 × g for 10 min at 4 °C, followed by resuspension of the particles in PBST. Next, biotinylated peptides were coupled to the particles by adding peptide (10 µL, 2 mM in MQ water) to AgNPs (500 µL), followed by incubation at RT for 30 min. The AgNPs were washed, 0.2 µm filtered and stored at 4 °C in the dark.

### Etching of AgNPs

To eliminate extracellular AgNPs, we exposed the live cells to a cell membrane-impermeable biocompatible etching solution (10 mM), 1: 1 solution of tripotassium hexacyanoferrate(III) (K_3_Fe(CN)_6_; CAS# 13,746–66-2, Sigma-Aldrich, Co., LLC) and sodium thiosulfate pentahydrate (Na_2_S_2_O_3_; CAS# 10,102–17-7, Sigma-Aldrich, Co., LLC) in PBS. After incubation with AgNPs, the cells were treated with the etching solution (400 µL, 10 mM) for 3 min at RT, followed by 2 washes with PBS. During etching, the AgNPs are dissolved by oxidation with hexacyanoferrate, and the Ag^+^ ions form a complex with the thiosulfate component. After the treatment only membrane-protected intracellular AgNPs remain intact and detectable.

### Cell culture

Prostate carcinoma cells (PPC1; ATCC) and melanoma cells (M21; ATCC) were grown as attached cultures in tissue culture-treated flasks (Corning Inc., USA) in high-glucose Dulbecco’s Modified Eagle Medium (DMEM; Lonza Ltd, Basel, Switzerland) with added 10% fetal bovine serum (FBS; Thermo Scientific Inc.), penicillin (Thermo Scientific Inc.) and streptomycin (Thermo Scientific Inc.).

Glioblastoma cells (U87-MG; ATCC) were grown as attached cultures in tissue culture-treated flasks (Corning Inc., USA) in high-glucose Dulbecco’s Modified Eagle Medium (DMEM; Lonza Ltd, Basel, Switzerland) containing 10% FBS (Thermo Scientific Inc.), 100 IU/mL penicillin (Thermo Scientific Inc.), 100 IU/mL streptomycin (Thermo Scientific Inc.), 1% sodium pyruvate (Sigma-Aldrich) and 1% non-essential amino acids (Sigma-Aldrich).

WT-GBM and VEGF-KO-GBM cell lines [55], which were a gift from Gabriele Bergers (Leuven, Belgium), were cultured in MEM with Earl's salts (Capricorn Scientific, Germany) supplemented with 100 IU/mL penicillin (Thermo Scientific Inc.), 100 IU/mL streptomycin (Thermo Scientific Inc.), 1% sodium pyruvate (Sigma-Aldrich), 0.01 M HEPES, 0.6% glucose (Applichem, USA) and 5% of heat-inactivated fetal bovine serum (GE Healthcare, UK). NCH421k cells [56], also a gift from G. Bergers, were cultivated in neurobasal medium complemented with B27 neurobasal supplement (Thermo Fisher Scientific, USA), 100 IU/mL of penicillin/streptomycin (Thermo Scientific Inc.), 0.4 mM Glutamax, 20 ng/ml basic FGF, 20 ng/ml EGF and 40 μg/ml of heparin (all Sigma-Aldrich, USA).

All cell lines were cultured at 37 °C in humidified atmosphere with 5% CO_2_. Before use, cells were rinsed with DPBS, dissociated with Cellstripper (Thermo Scientific Inc.), counted with a TC20™ Automated Cell Counter (Bio-Rad Laboratories AB, Sundyberg, Sweden), and diluted with supplemented medium or PBS as needed.

### In vitro experiments with AgNPs

Semiconfluent cells cultured on coverslips (d = 12 mm; Paul Marienfeld GmbH & Co. KG) were incubated with CF555-labeled AgNPs (1.5 nM final concentration) at 37 °C for 1 h. Optionally, prior to adding the AgNPs, the AgNPs were preincubated with 2 µL of uPA (#672,112, Sigma-Aldrich) reconstituted at 10,000 U/mL in MQ water per sample for 1 h at 37 °C in 1.5 mL Eppendorf tubes (Sigma-Aldrich). After incubation with the AgNPs and optional etching (see above), the cells were washed 3 times with 500 µL of DPBS, fixed with − 20 °C methanol (MeOH; Sigma-Aldrich) for 1 min, washed 2 times with DPBS, and counterstained with DAPI (500 µL, 1 µg/mL; Thermo Scientific Inc.). For confocal microscopy imaging, microscopy slides (76 × 26 mm, Glaswarenfabrik Karl Hecht GmbH & Co. KG) were coverslipped using an aqueous mounting medium Fluoromount-G (Electron Microscopy Sciences).

### Animal work

Athymic nude mice were purchased from the Tartu University Laboratory Animal Centre (Estonia). Female mice were used in all in vivo experiments if not stated otherwise. For induction of orthotopic GBM xenografts in nude mice, 7 × 10^5^ (WT-GBM, VEGF-KO-GBM) or 3 × 10^5^ (NCH421k, U87-MG) cells were implanted intracranially in the right striatum of the brain (coordinates: 2 mm laterally and 2 mm posteriorly from bregma and at 2.5 mm depth). Intracranial tumors were allowed to develop for 6–7 (WT-GBM), 12–14 (VEGF-KO-GBM, U87-MG), or ~ 30 (NCH421k) days before performing experiments, and mice were monitored for signs of tumor burden.

For AgNP and FAM-peptide biodistribution studies, tumor-bearing nude mice were i.v. injected with 100 μL of AgNPs (11.3 nM) or FAM-peptide (200 μg; TAG Copenhagen, Denmark). After 3 h (AgNP) or 1 h (FAM-peptide) of circulation, the mice were anesthetized with intraperitoneally (i.p.) injected 350 μL of 0.1 mg/kg dexmedetomidine and 75 mg/kg ketamine dissolved in saline, and perfused with PBS. The brain, tumor, lungs and liver were collected, frozen in OCT (Leica Camera AG, Germany), sectioned with a Leica CM1520 (Leica Camera AG, Germany) at 15 μm (for immunostaining) onto Superfrost Plus slides (Thermo Fisher, USA), and dried in a vacuum desiccator (Novus DN 200, Buch & Holm, Germany).

For LA-ICP-MS analysis, the ^107^AgNPs (functionalized with PL3, SKLG or PL3uCendR peptide) and control ^109^AgNPs (blocked with biotin) were mixed in equimolar ratio and i.v. injected into orthotopic WT-GBM-bearing nude mice (Envigo). In total, 100 μL of AgNPs (11.3 nM) was injected per mouse. After 3 h of circulation, the mice were anesthetized with intraperitoneally (i.p.) injected 350 μL of 0.1 mg/kg dexmedetomidine and 75 mg/kg ketamine dissolved in saline, and perfused with PBS. The brain, tumor, lungs and liver were collected, frozen in OCT (Leica Camera AG, Germany), sectioned with a Leica CM1520 (Leica Camera AG, Germany) at 30 μm onto Superfrost Plus slides (Thermo Fisher, USA), and dried in a vacuum desiccator (Novus DN 200, Buch & Holm, Germany).

### Immunohistochemistry and immunofluorescence analyses

For uPA expression staining on 2D cultured cells, the cells were fixed in − 20 °C MeOH for 1 min, washed with PBS and blocked with 300 μL of 5% blocking solution containing 0.05% Tween-20 (Sigma-Aldrich, USA), 5% fetal bovine serum (FBS), 5% bovine serum albumin (BSA), and 5% goat serum (all from GE Healthcare, USA) in PBS for 1 h. The sections were incubated for 1 h at RT with 300 μL 0.9 mg/mL polyclonal rabbit anti-uPA IgG (#17,968–1-AP, Proteintech Group, Inc., USA) as the primary antibody which was diluted 1: 100 with 1% blocking solution. After washing with PBST, sections were incubated for 30 min at RT with 250 μL of 2 mg/mL Alexa 647-conjugated goat anti-rabbit IgG (#A21245, Invitrogen, USA) as the secondary antibody, which was diluted 1: 1000 in 1% blocking solution. After washing sequentially with PBST and PBS, nuclei were counterstained with 1 μg/mL DAPI (Sigma-Aldrich, USA) in PBS.

For immunofluorescence analysis, cryosections (15 μm) on Superfrost Plus slides were fixed in − 20 °C MeOH for 2 min, washed with PBS, and blocked with 300 μL of 5% blocking solution containing 0.05% Tween-20 (Sigma-Aldrich, USA), 5% FBS, 5% BSA, and 5% goat serum (all from GE Healthcare, USA) in PBS for 1 h. The sections were incubated for 1 h at RT with 250 μL of 0,5 mg/mL polyclonal rat anti-mouse CD31 (#553,370, BD Biosciences, USA), 250 μL of 1.2 mg/mL polyclonal rabbit anti-NRP-1 (in-house), 1.4 mg/mL polyclonal rabbit anti-TNC (in-house) or 0.9 mg/mL polyclonal rabbit anti-uPA IgG (#17,968–1-AP, Proteintech Group, Inc., USA) as primary antibodies, which were diluted 1: 200 with 1% blocking solution. After washing with PBST, sections were incubated for 30 min at RT with 250 μL of 2 mg/mL Alexa 647-conjugated goat anti-rabbit IgG (#A21245, Invitrogen) or Alexa 546-conjugated goat anti-rat (#A11081, Invitrogen, USA) as the secondary antibody, which was diluted 1: 1000 in 1% blocking solution. After washing sequentially with PBST and PBS, nuclei were counterstained with 1 mg/mL DAPI (Sigma-Aldrich, USA) in PBS.

For hematoxylin & eosin (H&E) staining, cryosections (15 μm) on Superfrost Plus slides were air-dried at RT, fixed in − 20 °C MeOH for 2 min, and rinsed in MQ. Slides were stained with Mayer’s hematoxylin (#180,210, Reagena, Finland) for 5 min, rinsed with running cold tap water for 5 min, stained with 0,5% eosin (#230,251, Sigma-Aldrich, USA) in 95% ethanol (EtOH, Sigma-Aldrich, USA) with 0,5% of acetic acid (Sigma-Aldrich, USA) for 5 min, and rinsed again with cold tap water and MQ for 5 min. After air-drying the sections, they were mounted with 130 μL of Depex (Merck Millipore, USA), and imaged with the Aperio VERSA 10 (Leica Biosystems, Germany).

For 3,3’-diaminobenzidine (DAB) staining, cryosections (15 μm) on Superfrost Plus slides were air-dried at RT, fixed in 4% paraformaldehyde (PFA; Sigma-Aldrich, USA) for 20 min, permeabilized with 0.2% Triton X-100 (AppliChem PanReac, USA) for 15 min. Sections were washed with 0.1% Tween 20 (Sigma-Aldrich, USA) in PBS and blocked with 3% H_2_O_2_ (Sigma-Aldrich, USA) in PBS on a rocking platform (SIA BioSan, Latvia) for 30 min at RT. After washing with PBS, sections were blocked with 5% heat-inactivated normal donkey serum (GE Healthcare) and 5% BSA (GE Healthcare), 0.3 M glycine in PBS for 60 min at RT. Sections were incubated overnight at + 4 °C with 250 µL of 1: 200 diluted 1 mg/mL rabbit anti-FAM IgG (#A889, Invitrogen, USA) in blocking buffer as the primary antibody. After washing with PBS, sections were incubated at RT for 1 h with 250 µL of 1: 200 diluted donkey-anti-rabbit HRP (#406,401, Biolegend, USA) as the secondary antibody. After washing with PBS, sections were incubated with 500 µL of ImmPACT DAB (#SK-4105, Vector Laboratories) for 1 min at RT. After washing with MQ for 30 min, sections were air-dried, mounted with 130 μL Depex (Merck Millipore, USA), and imaged with the Aperio VERSA 10 (Leica Biosystems, Germany).

### Microscopy

For H&E and DAB staining imaging, parallel tissue sections were immunostained with H&E and DAB as described above. Sections were imaged with a 10 × objective (HC PL FLUOTAR 10 × /0.32; Leica Microsystems, Germany) mounted to an Aperio VERSA 10 Brightfield, Fluorescence FISH Digital Pathology Scanner (Leica Biosystems, Germany). Images were analyzed with Aperio ImageScope v. 12.4.3.5008 software (Leica Biosystems Pathology Imaging, Germany).

Confocal imaging was performed using an Olympus FV1200MPE confocal microscope (Olympus Europa SE & Co. KG), and images were analyzed with FluoView FV10-ASW 4.0 software (Olympus Europa SE & Co. KG).

### LA-ICP-MS analysis

Laser ablation inductively coupled plasma mass spectrometry (LA-ICP-MS) analysis of the tissue samples was performed using Agilent 8800 ICP-MS/MS coupled to a Cetac LSX-213 G2 + laser ablation unit equipped with HelEx II ablation cell and connected using ARIS (Aerosol Rapid Introduction System) sample introduction system. The system was tuned using NIST 610 glass. Ablation was performed as line scans using 65 μm square spot, scan speed of 260 μm/s, 20 Hz and fluence of 12 J/cm^2^. All tissue samples were fully ablated using 65 μm spacing of ablation lines. ICP-MS was operated in single quad mode. Data collection was performed in TRA mode with dwell times of 9.5 ms on mass ^13^C and 14 ms on mass ^107^Ag and ^109^Ag corresponding to a total duty cycle of 50 ms. Data reduction of LA-ICP-MS raw data was performed using Iolite v3.62 and Microsoft Excel. Median with MAD error 2 SD outlier reject was used for data selection in Iolite. To remove non-tissue areas in the analytical data, signal masking based on ^13^C signal was employed. 2D ^109^Ag/^107^Ag signal ratio maps were then constructed using Iolite v3.62 and exported as numeric maps for statistical analysis.

### Statistical analysis

To assess statistical significance, one- or two-way analysis of variance (ANOVA) with Tukey or Dunnett post-hoc test, as appropriate, was performed using GraphPad Prism Software v. 10.1.2 (GraphPad Software, LLC, CA, USA).

### Artwork and illustrations

The graphical abstract as well as Figs. 1a and 4a were created with BioRender.com software in accordance with the BioRender’s Academic License to Allan Tobi. Microsoft Office PowerPoint was used to create the figure panels.

## Supplementary Information

Below is the link to the electronic supplementary material.

Supplementary Material 1 (PDF 4.86 MB)

## Data Availability

The datasets generated during and/or analyzed during the current study are available from the corresponding author on reasonable request.
