# Supplementary material for: Protease-activated CendR peptides targeting tenascin-C: mitigating off-target tissue accumulation
Source: Drug Deliv Transl Res. 2024 Jul 16;14(10):2945–61. doi: 10.1007/s13346-024-01670-2 (PMC11384632; doi:10.1007/s13346-024-01670-2)
Supplement: Supplementary file 1 — Supplementary Material 1 (PDF 4.86 MB) [file 13346_2024_1670_MOESM1_ESM.pdf]

Drug Delivery and Translational Research

Supplementary material

Protease-activated CendR peptides targeting tenascin-C: mitigating off-target tissue accumulation

Allan Tobi[1], Maarja Haugas[1], Kristina Rabi[1], Jhalak Sethi[1], Kristina Põšnograjeva[1], Karlis Pleiko[1], Päärn Paiste[2], Toomas Jagomäe[3], Prakash Lingasamy[4], Tambet Teesalu[1,5]*

[1] Laboratory of Precision and Nanomedicine, Institute of Biomedicine and Translational Medicine, University of Tartu, Ravila 14b, 50411 Tartu, Estonia.

[2] Department of Geology, Institute of Ecology and Earth Sciences, University of Tartu, Ravila 14a, 50411 Tartu, Estonia

[3] Laboratory Animal Centre, Institute of Biomedicine and Translational Medicine, University of Tartu, Ravila 14b, 50411 Tartu, Estonia.

[4] Competence Centre on Health Technologies, Teaduspargi 13, 50411 Tartu, Estonia.

[5] Materials Research Laboratory, University of California, Santa Barbara, CA 93106, USA.

*Corresponding author: T. Teesalu, Laboratory of Precision and Nanomedicine, Institute of Biomedicine, University of Tartu, Ravila 14b, 50411 Tartu, Estonia. E-mail address: tambet.teesalu@ut.ee.

Table S1 Top 10 peptide sequences from phage display results based on enrichment ratio.

	+uPA			–uPA			Enrichment		Enrichment
Peptide	R1	R2	R3	R1	R2	R3	+uPA	–uPA	+uPA/–uPA
AGRGRLVRPTEF	17	73	505	0	0	1	252.5	0.5	505.0
AGRGRLVRADVR	16	65	252	2	4	1	84.0	0.3	252.0
AGRGRLVRSKLG	18	28	189	3	2	1	189.0	1.0	189.0
AGRGRLVRDDSN	6	24	125	7	3	1	62.5	0.5	125.0
AGRGRLVRTPV	2	9	94	0	5	1	47.0	0.5	94.0
AGRGRLVRAPDV	5	8	75	5	1	1	15.0	0.2	75.0
AGRGRLVRDDEV	3	6	74	1	1	1	74.0	1.0	74.0
AGRGRLVRSPDR	3	4	62	0	0	1	6.9	0.1	62.0
AGRGRLVRVEKG	2	4	46	0	0	1	46.0	1.0	46.0
AGRGRLVREASR	2	5	158	2	1	4	52.7	1.3	39.5

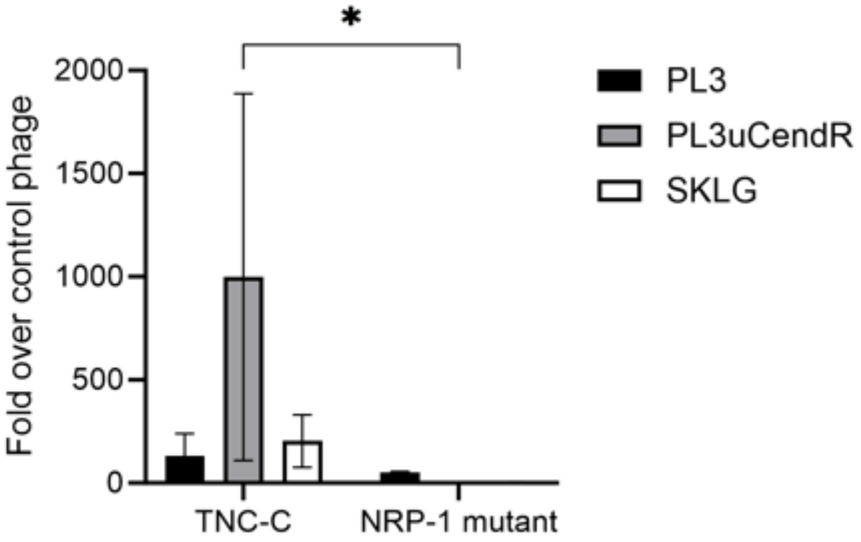

Fig. S1 Peptide-phage retains binding to TNC-C. Ni-NTA magnetic agarose beads were coated with recombinant C-domain of TNC (TNC-C) or recombinant mutated b1 domain of NRP-1 (NRP1 mutant) as the negative control, incubated with peptide-phages, washed, eluted, titered. Results are displayed as fold over control G7 phage. Error bars show standard deviation (SD) (*n* = 3).

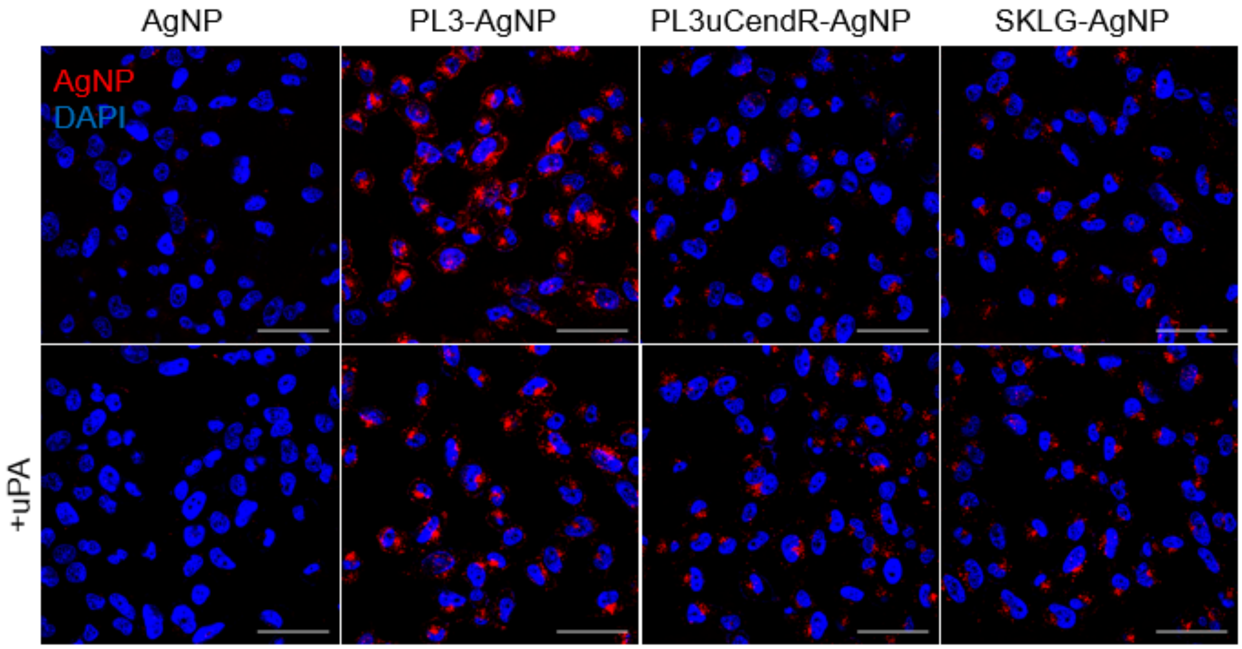

Fig. S2 Binding of CendR peptide-AgNPs in NRP-1-positive PPC1 cells. Prostate carcinoma (PPC1) cells were grown as a 2D culture, incubated with CF555-labeled AgNPs (red) optionally pretreated with uPA, washed, fixed with –20 °C MeOH, counterstained with DAPI (blue; nuclei), and imaged. Representative images are shown (n = 3). Scale bar: 100 µm.

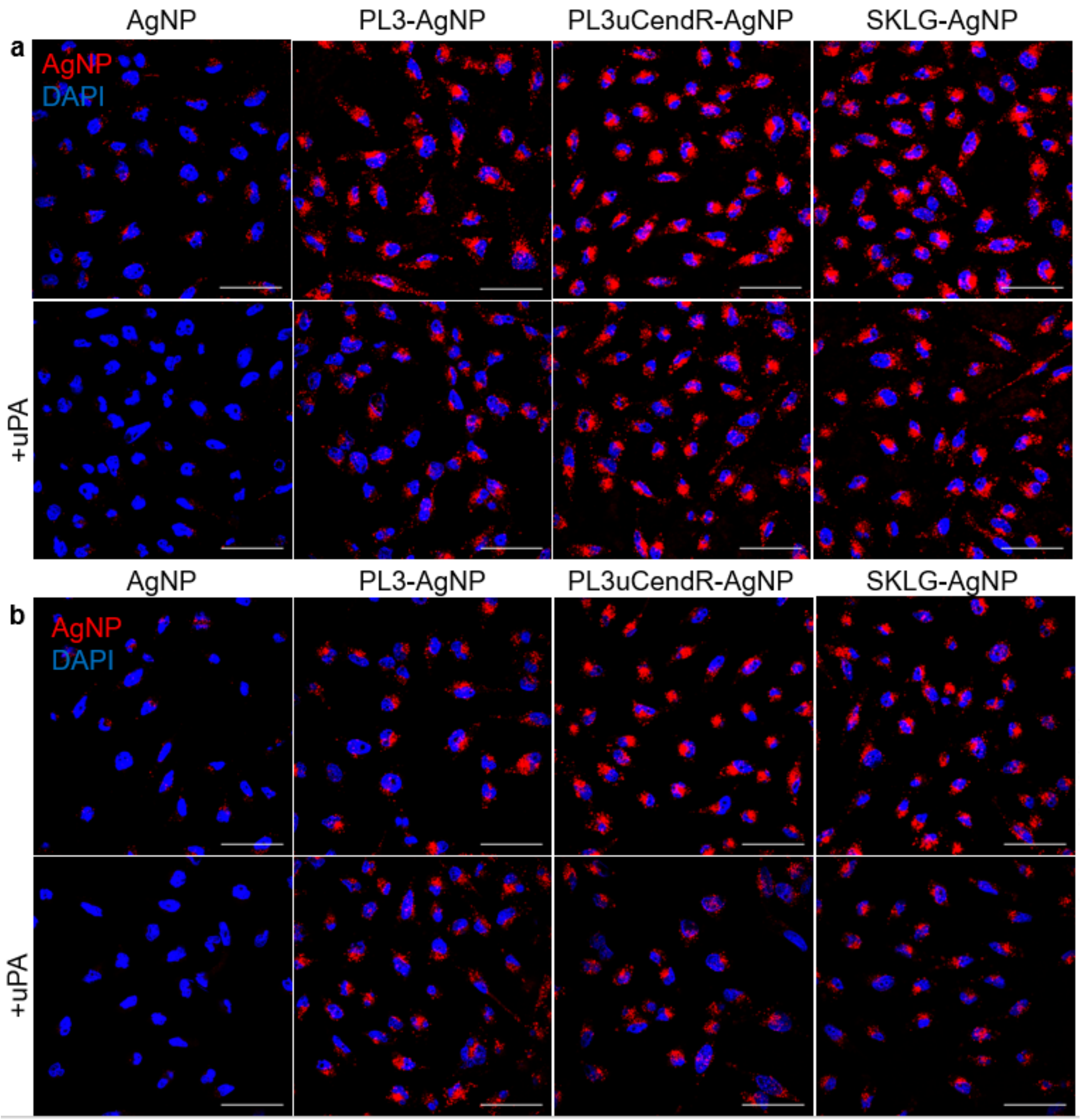

Fig. S3 Binding and internalization of CendR peptide-AgNPs in NRP-1- and TNC-positive U87-MG cells. (**a**) Binding of CendR peptide-AgNPs. U87-MG cells were grown as a 2D culture, incubated with CF555-labeled AgNPs (red) optionally pretreated with uPA, washed, fixed with –20 °C MeOH, counterstained with DAPI (blue; nuclei), and imaged. (**b**) Internalization of CendR peptide-AgNPs. U87-MG cells were treated with a membrane-impermeable AgNP dissolving solution before fixation to visualize internalized nanoparticles. Representative images are shown (n = 3). Scale bar: 100 µm.

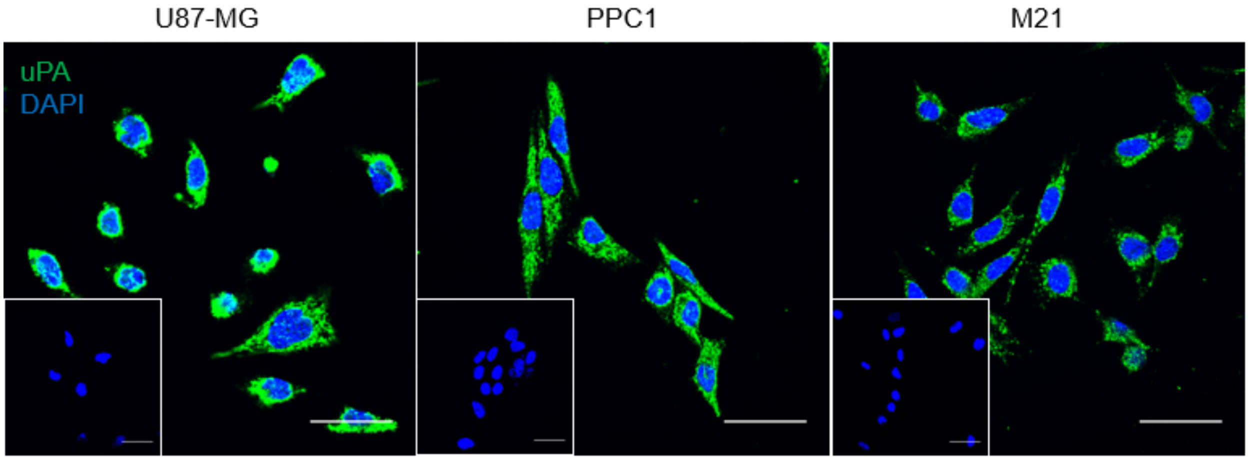

Fig. S4 Expression of uPA in (**a**) U87-MG, (**b**) PPC1, and (**c**) M21 cells. Cancer cells were grown as a 2D culture, fixed with –20 °C MeOH, stained with primary polyclonal rabbit anti-uPA antibody and secondary goat anti-rabbit CF647-labeled antibody (green), washed, counterstained with DAPI (blue; nuclei), and imaged. Inserts show secondary antibody control. Representative images are shown (n = 3). Scale bar: 100 µm.

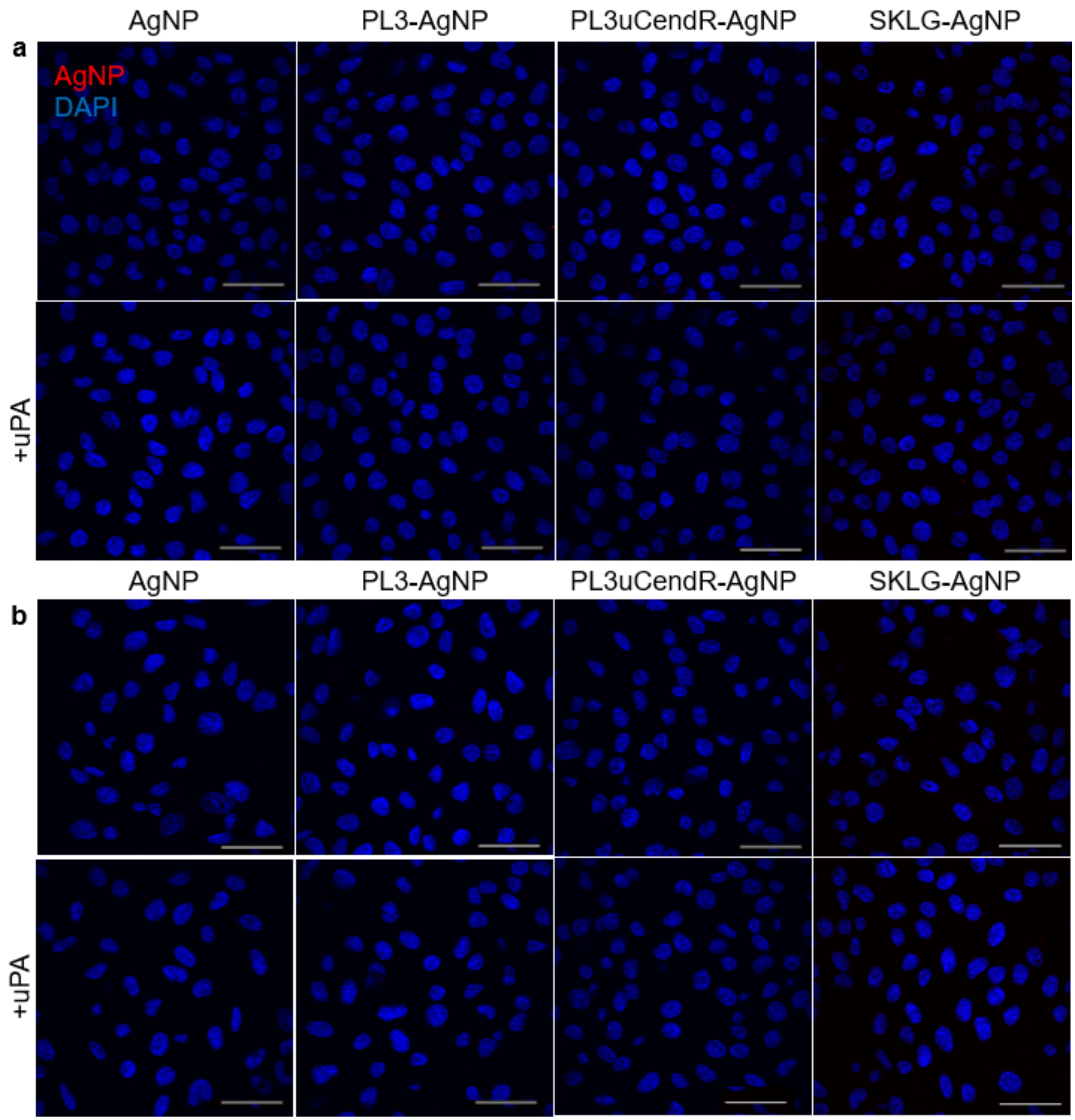

Fig. S5 (**a**) Binding and (**b**) internalization of CendR peptide-AgNPs in NRP-1- and TNC-negative M21 cells. M21 cells were grown as a 2D culture, incubated with CF555-labeled AgNPs (red) optionally pretreated with uPA, washed, fixed with –20 °C MeOH, counterstained with DAPI (blue; nuclei), and imaged; for internalization (b), cells were etched with a membrane-impermeable AgNP dissolving solution before fixation. Representative images are shown (n = 3). Scale bar: 100 µm.

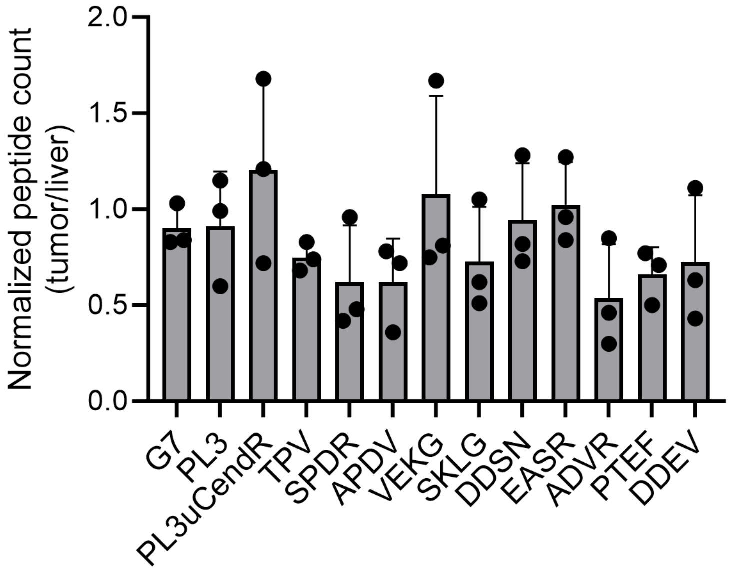

Fig. S6 Auditioning candidate peptides by *in vivo* peptide phage playoff. Phages expressing peptide of interest or control peptide mixed in equimolar ratio and injected i.v. into WT-GBM-bearing female nude mice. After 30 min, mice were anesthetized and perfused, organs were harvested, homogenized, and tissue lysates were amplified, purified and sequenced with HTS. Results are shown as peptide count in WT-GBM tumor over liver normalized to control G7 phage. Error bars show standard deviation (SD), scatter symbols individual measurements ($n = 3$).

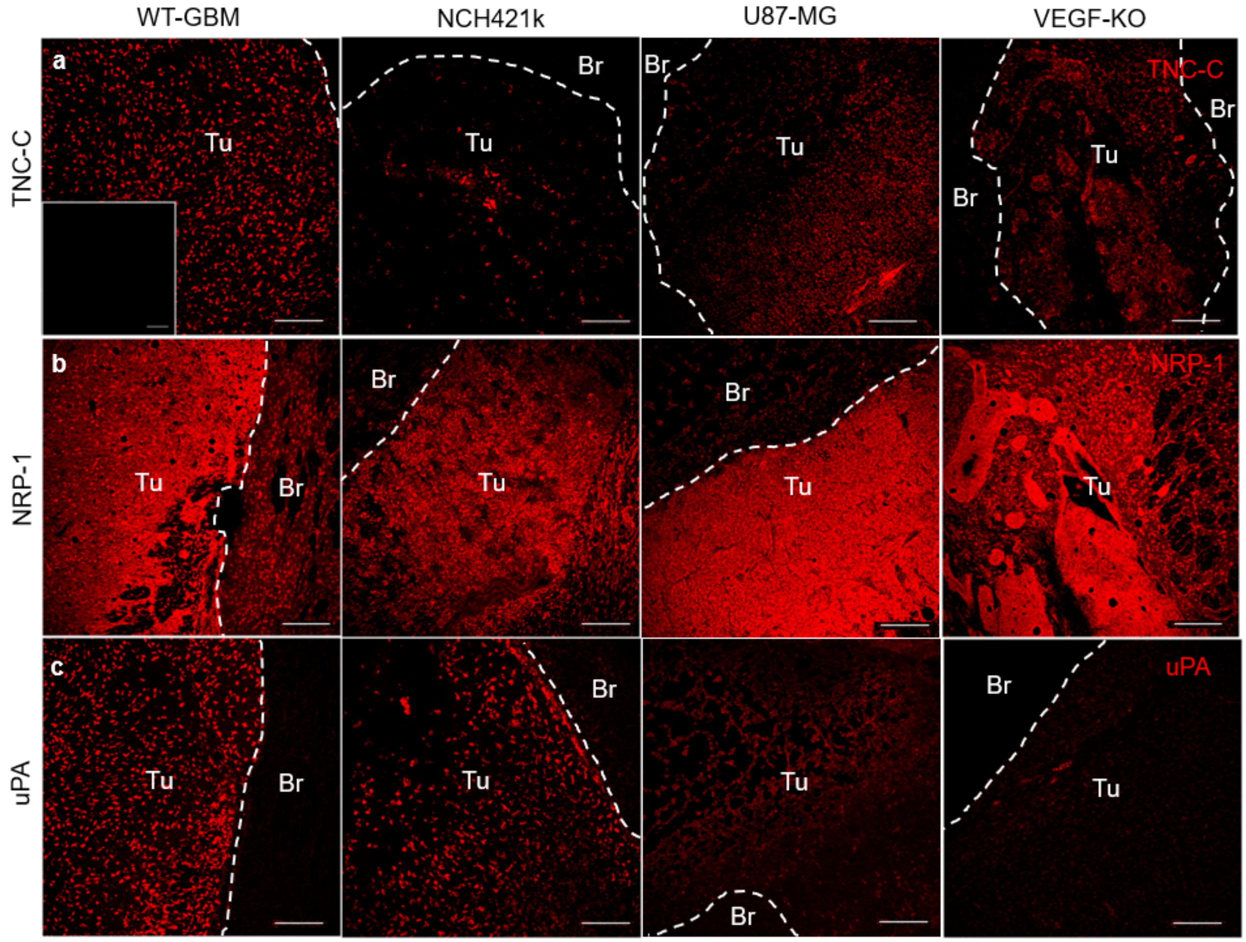

Fig. S7 Expression of (**a**) TNC-C, (**b**) NRP-1, and (**c**) uPA in WT-GBM, NCH421k, U87-MG, and VEGF-KO orthotopic tumor lesions in brains of nude mice. Mouse brains with orthotopic tumors were sectioned, permeabilized and fixed with –20 °C MeOH, blocked with a 5% blocking solution, stained with primary polyclonal rabbit anti-TNC-C, anti-NRP-1 or anti-uPA antibody and secondary goat anti-rabbit CF647-labeled antibody (red), washed, and imaged. Insert in (a) shows secondary antibody control. White dashed lines outline tumor lesions: Tu = tumor, Br = brain. Representative images are shown (n = 3). Scale bar: 200 µm.

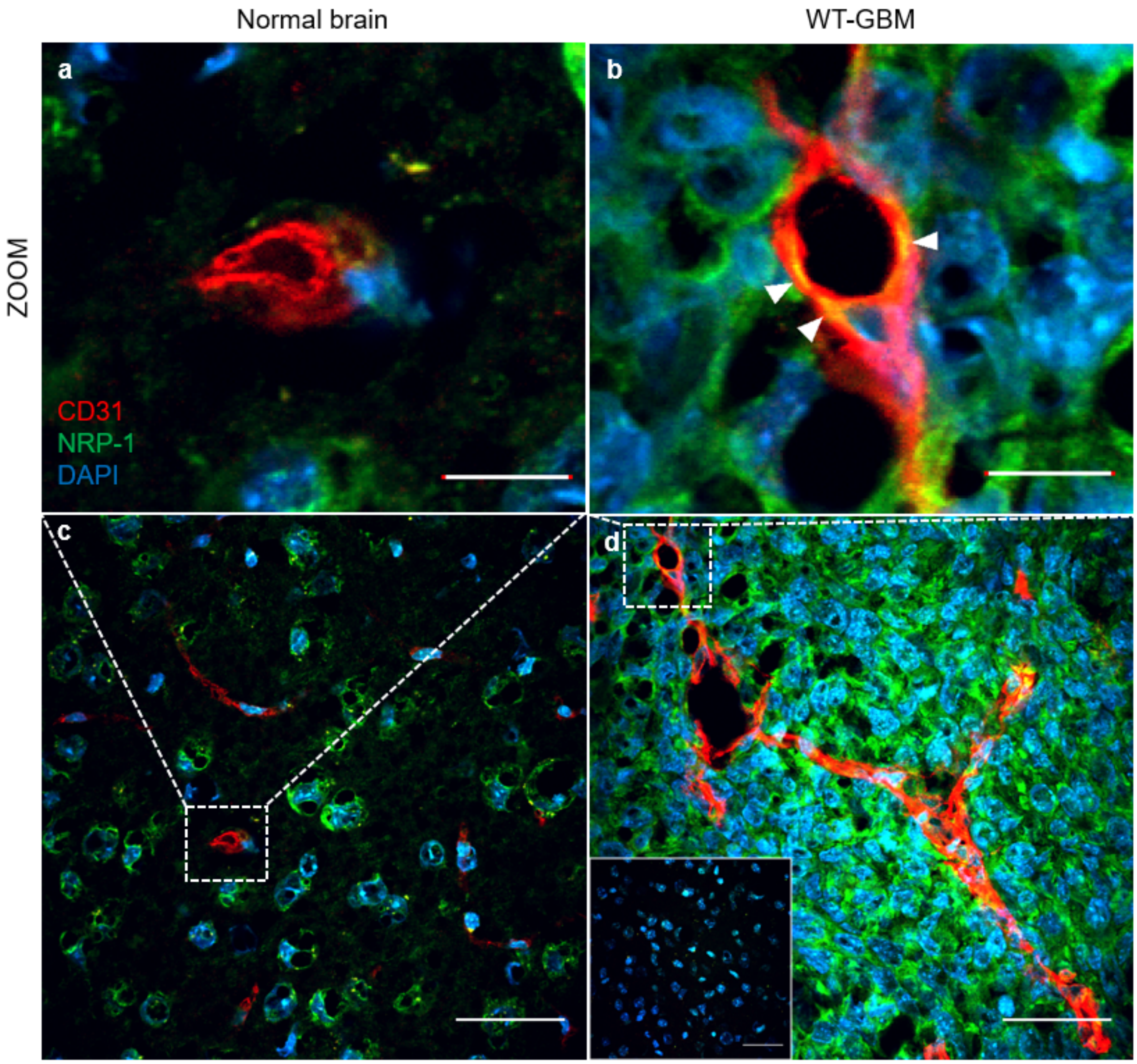

Fig. S8 Colocalization of NRP-1 and CD31-positive blood vessels in (**a, c**) the tumor-free brain region and (**b, d**) WT-GBM orthotopic tumor lesions in mice. Mouse brains with orthotopic tumors were sectioned, permeabilized and fixed with –20 °C MeOH, blocked with a 5% blocking solution, stained with primary polyclonal rabbit anti-NRP-1 or rat anti-CD31 antibody and secondary goat anti-rabbit AF647-labeled (green) or anti-rat AF546 antibody (red), respectively, washed, and imaged. Insert in (d) shows secondary antibody control. Arrowheads point to colocalization. Representative images are shown (n = 3). Scale bars: 25 µm (a, b) and 100 µm (c, d).

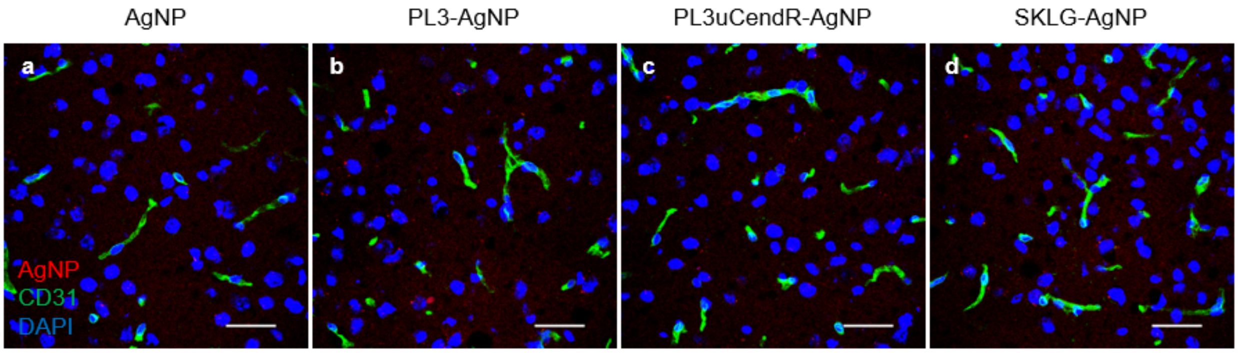

Fig. S9 Accumulation of AgNPs in tumor-free regions of the brains of tumor-bearing mice. Mice were i.v. injected with CF555-labeled AgNPs (red). After 3 h of circulation the mice were anesthetized and perfused. Organs were harvested, sectioned, immunostained with anti-CD31 antibody (green; blood vessels), counterstained with DAPI (blue; nuclei). Representative images are presented (*n* = 3). Scale bar: 100 μm.

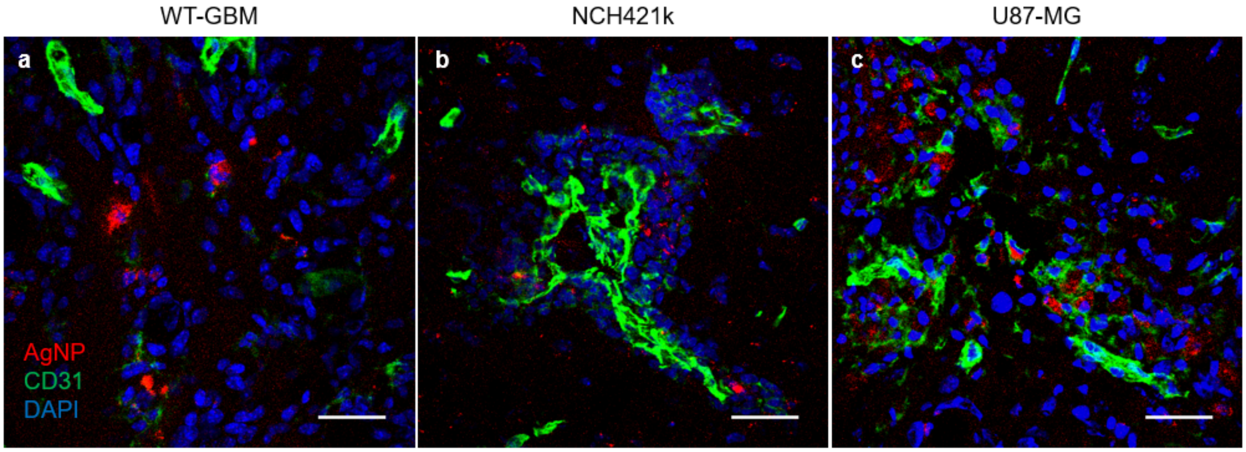

Fig. S10 High accumulation areas of control AgNPs in (**a**) WT-GBM, (**b**) NCH421k and (**c**) U87-MG GBM models. Orthotopic GBM-bearing nude mice were i.v. injected with CF555-labeled AgNPs (red). After 3 h of circulation the mice were anesthetized and perfused. Organs were harvested, sectioned, immunostained with anti-CD31 antibody (green; blood vessels), counterstained with DAPI (blue; nuclei). Representative images are presented (*n* = 3). Scale bar: 100 μm.

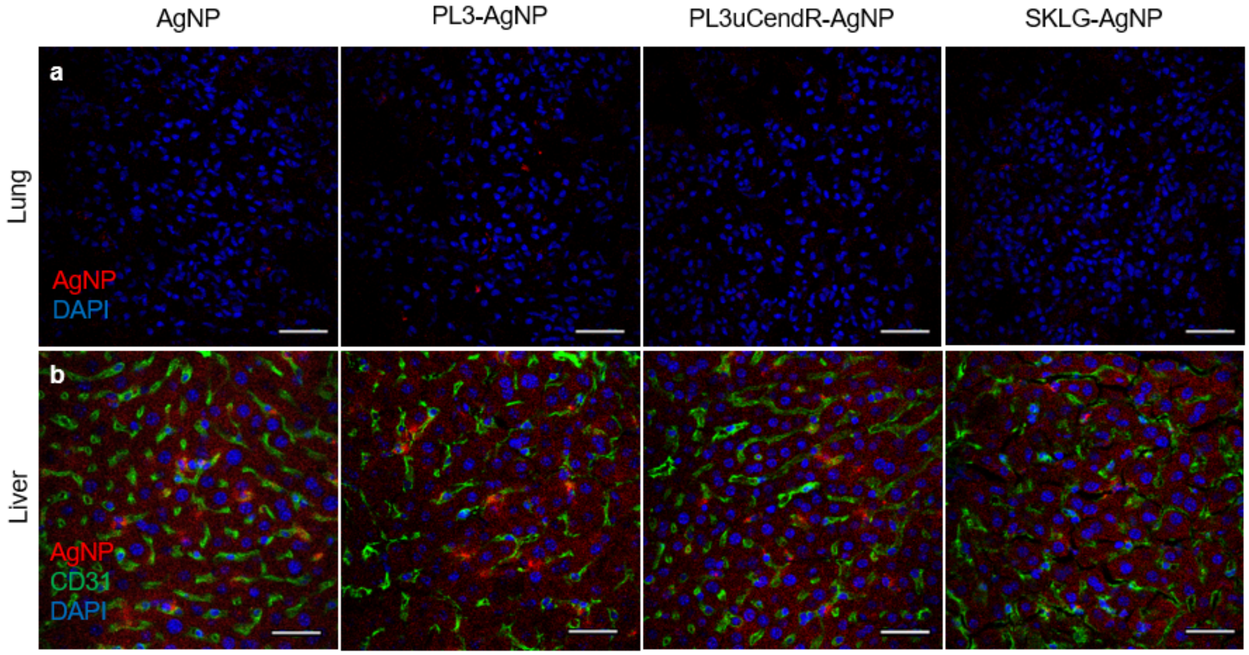

Fig. S11 Biodistribution of AgNPs in (**a**) pulmonary and (**b**) liver tissues of mice. Orthotopic WT-GBM-bearing nude mice were i.v. injected with CF555-labeled AgNPs (red). After 3 h of circulation the mice were anesthetized and perfused. Organs were harvested, sectioned, immunostained with anti-CD31 antibody (green; blood vessels), counterstained with DAPI (blue; nuclei). CD31 channel in (a) was omitted for clarity. Representative images are presented (n = 3). Scale bar: 100 µm.

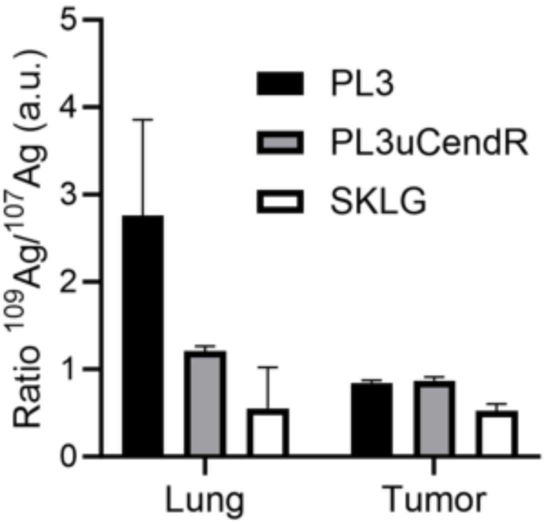

Fig. S12 Internally-controlled LA-ICP-MS-based quantitative mapping of isotopically labeled AgNPs. Orthotopic WT-GBM-bearing nude mice were i.v. injected with an equimolar mixture of peptide-^{109}AgNPs and biotin-blocked control ^{107}AgNPs. After 3 h of circulation the mice were anesthetized, and perfused. Organs were harvested, sectioned, and subjected to laser ablation inductively coupled plasma mass spectrometry (LA-ICP-MS) analysis. Error bars show standard deviation (SD) (n = 3); a.u. = arbitrary units.
